# Self‐Induced Mode‐Locking in Electrically Pumped Far‐Infrared Random Lasers

**DOI:** 10.1002/advs.202206824

**Published:** 2023-01-27

**Authors:** Alessandra Di Gaspare, Valentino Pistore, Elisa Riccardi, Eva A. A. Pogna, Harvey E. Beere, David A. Ritchie, Lianhe Li, Alexander Giles Davies, Edmund H. Linfield, Andrea C. Ferrari, Miriam S. Vitiello

**Affiliations:** ^1^ NEST CNR – Istituto Nanoscienze and Scuola Normale Superiore Piazza San Silvestro 12 Pisa 56127 Italy; ^2^ CNR – Istituto di Fotonica e Nanotecnologie Piazza Leonardo da Vinci 32 Milano 20133 Italy; ^3^ Cavendish Laboratory University of Cambridge Cambridge CB3 0HE UK; ^4^ School of Electronic and Electrical Engineering University of Leeds Leeds LS2 9JT UK; ^5^ Cambridge Graphene Centre University of Cambridge Cambridge CB3 0FA UK

**Keywords:** graphene, random lasers, terahertz

## Abstract

Mode locking, the self‐starting synchronous oscillation of electromagnetic modes in a laser cavity, is the primary way to generate ultrashort light pulses. In random lasers, without a cavity, mode‐locking, the nonlinear coupling amongst low spatially coherent random modes, can be activated via optical pumping, even without the emission of short pulses. Here, by exploiting the combination of the inherently giant third‐order *χ* ^(3)^ nonlinearity of semiconductor heterostructure lasers and the nonlinear properties of graphene, the authors demonstrate mode‐locking in surface‐emitting electrically pumped random quantum cascade lasers at terahertz frequencies. This is achieved by either lithographically patterning a multilayer graphene film to define a surface random pattern of light scatterers, or by coupling on chip a saturable absorber graphene reflector. Intermode beatnote mapping unveils self‐induced phase‐coherence between naturally incoherent random modes. Self‐mixing intermode spectroscopy reveals phase‐locked random modes. This is an important milestone in the physics of disordered systems. It paves the way to the development of a new generation of miniaturized, electrically pumped mode‐locked light sources, ideal for broadband spectroscopy, multicolor speckle‐free imaging applications, and reservoir quantum computing.

## Graphical abstract

Locking of low spatial coherence modes in surface‐emitting electrically pumped random quantum cascade lasers at terahertz frequencies, by either integrating a multilayer graphene film into the random pattern of light scatterers or by coupling a graphene reflector on chip. Self‐mixing intermode spectroscopy reveals phase‐locked random modes.

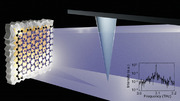

## 1 Introduction

Random lasers (RLs),^[^ ^1^, ^2^ ^]^ comprising a random distribution of discrete light scattering elements embedded in a gain medium, strongly differ from conventional lasers.^[^ ^1^, ^2^ ^]^ RLs include an optically active medium, providing gain, and a disordered arrangement^[^ ^3^, ^4^ ^]^ of scatterers, providing the required refractive index change and feedback mechanism that lead to light amplification by stimulated photon emission. The emitted photons can be amplified and coherently interact many times in the gain medium, resulting in a rich interference scheme possessing high temporal coherence, due to the high interaction length associated with the photon random walk in the disordered medium, and low spatial coherence,^[^ ^5^ ^]^ due to the coexistence of randomly distributed lasing modes with distinct and spatially separated wave fronts.^[^ ^5^ ^]^

Mode engineering in RLs requires a deep knowledge of the core physical mechanisms determining photon emission and mode interaction above threshold. While in standard lasers the latter process is highly nonlinear, in RLs it is more difficult to assess the nature of this interaction.^[^ ^6^ ^]^ Unlike RLs with self‐assembled resonators in the IR range,^[^ ^7^ ^]^ disordered electrically pumped quantum cascade lasers (QCLs), such as quasi‐crystals^[^ ^8^, ^9^ ^]^ or hyperuniform QCLs,^[^ ^10^ ^]^ are characterized by reduced density fluctuations compared to a purely random system.^[^ ^11^ ^]^ This definition includes all periodic, such as photonic crystals,^[^ ^12^ ^]^ and aperiodic,^[^ ^10^ ^]^ quasi‐crystal lasers.^[^ ^13^ ^]^ These designs enable engineering the emission properties of a disordered distribution in an amplifying medium.

The simultaneous presence of structural disorder and nonlinearity, however, makes RLs ideal for investigating exotic phenomena connected with the specific nature of the mode interaction, such as chaos,^[^ ^14^ ^]^ non‐Gaussian statistics,^[^ ^15^ ^]^ non‐Gaussian complexity,^[^ ^16^ ^]^ Anderson localization,^[^ ^17^ ^]^ and mode locking.^[^ ^18^ ^]^

Mode‐locking is a nonlinear phenomenon, occurring when the interaction between laser modes leads to their locking in phase. In standard lasers, stable self‐starting synchronous oscillation of electromagnetic modes in a cavity occurs spontaneously in the presence of a saturable absorber (SA), promoting the generation of ultrashort pulses.^[^ ^19^, ^20^ ^]^ However, in RLs a detailed understanding of the origin and strength of the correlation mechanism and the related number of cross‐correlated interacting optical modes is missing. A first intuitive condition for nonlinear interaction among laser modes is their spatial overlap,^[^ ^21^ ^]^ which is, however, neither a sufficient condition for mode‐locking, nor does provide direct information on the coupling strength. The structure of the modes spatial distribution in most RLs is hard to determine, making identification and quantitative analysis of the interaction parameters difficult.

A variety of approaches has been adopted to control RL modes^[^ ^22^ ^]^, such as tuning of the scatterer statistic arrangement and concentration,^[^ ^23^ ^]^ external cavity configurations,^[^ ^8^ ^]^ or the reshaping of their spectral emission via adaptive optical pumping.^[^ ^24^ ^]^

Spontaneous mode‐locking in RLs was theoretically predicted.^[^ ^18^, ^25^ ^]^ The complex tunable optical pumping of a laser dye^[^ ^25^ ^]^ was exploited to promote the transition of RL modes from a resonant feedback regime, characterized by few uncorrelated sharp peaks, to an incoherent one, compatible with the emission of a smooth broader spectrum with a high degree of correlation.^[^ ^18^ ^]^ Quasi mode‐locking in a coherent feedback fiber RLs was demonstrated by optical pumping of a partially disordered linear cavity, formed between a point reflector and a random distributed fiber Bragg grating array with an inserted graphene saturable absorber (GSA).^[^ ^26^ ^]^ Self‐starting mode locking in RLs based on a layer of GaAs powder was also demonstrated.^[^ ^6^ ^]^ Optical pumping in the visible range was exploited to induce four‐mode intensity correlations that led to frequency matching as an effect of *χ* ^(3)^ nonlinearity,^[^ ^6^ ^]^ without any external modulator or SAs, but only exploiting the intrinsic randomness^[^ ^6^ ^]^.

Disordered RLs, originally conceived in optically pumped suspended microparticle laser dye,^[^ ^27^ ^]^ fine powders,^[^ ^28^ ^]^ or bone tissues,^[^ ^29^ ^]^ were reported in the technologically appealing mid‐IR (9–10.5 µm)^[^ ^30^ ^]^ and terahertz (THz) (2.8–3.5 THz)^[^ ^8^, ^23^, ^31^, ^32^, ^33^ ^]^ frequency ranges, exploiting electrically pumped QCLs embedded in either one dimensional, 1d^[^ ^31^ ^]^, or two dimensional, 2d^[^ ^23^ ^]^, photonic resonators. Through a combination of different designs^[^ ^23^ ^]^ and architectures,^[^ ^8^, ^24^, ^25^, ^26^, ^27^ ^]^ large power outputs (80 mW peak power) and rich (up to 13 modes) multimode emission, over the entire optical bandwidth of the designed QC structure (450 GHz) in both pulsed and continuous‐wave (CW)^[^ ^14^, ^32^ ^]^ were achieved. Broadband spectroscopic and multicolor speckle‐free imaging applications can benefit from the development of mode‐locked RLs across the THz. However, no exploration of mode‐locking effects in such a class of electrically pumped sources was reported to date, to the best of our knowledge.

**Figure 1:**
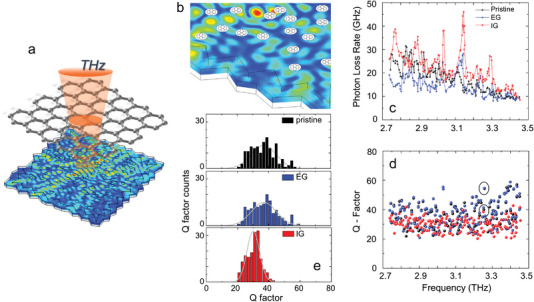
Graphene integration in random quantum cascade lasers. a) Device schematics of the graphene coupled/integrated 2d random QCL. The random resonator, with a total area ∼0.076 mm^2^ (filling fraction 10%), comprises a 2d arrangement of *N* = 99 holes randomly distributed on a square area with size *L* = 275 µm, and irregular shaped borders coated with a 10‐nm‐thick layer of lossy Cr to suppress geometric cavity resonances. The photonic filling factor (FF), defined as *r*/*a*, where *r* = 5 µm is the hole radius and $a=L/\sqrt{N}$, is 18%. The scattering efficiency in the present device can be estimated within the Mie scattering theory, by assuming the scatterers as cylindrical holes.^[^ ^51^ ^]^ The transport mean free path for the random modes is 10.4 µm. b) Schematic integration of multilayer graphene (MLG) inside the scatterer holes on the top emitting surface of the random resonator. The random scatterers arrangement is realized by transferring a pattern of random holes to the top Au contact and to the high‐doping GaAs top cladding. The color scheme in (a) and (b) represents the electric field distribution inside the resonator at the ∼3.25 THz eigenmodes highlighted with two black circles in (d), calculated at the half height of the mesa (5 µm below the top surface). c) Photon loss rate and d) quality factors (*Q*_tot_) calculated for the 2d random resonator in (a), for 3 configurations: pristine (black), EG mirror (blue), and IG (red). In the EG simulation, the graphene mirror is placed ∼50 µm from the laser top surface, mimicking the experiment. The two quantities are reported as a function of the specific computation eigenmodes. The Q‐factors are extracted from the simulation. The photon loss rate is calculated as the ratio between total power output, retrieved by integrating the electromagnetic (EM) power over the top surface, and total internal electric field energy, upon integration of EM energy over the resonator volume. e) Frequency count histograms of the Q‐factors of (d), for pristine (top, black), EG (center, blue), and IG (bottom, red) cases, to illustrate statistical Q‐values distributions. The gray curves are the standard Gaussian‐like distribution in the 3 cases, calculated by using mean value and standard deviation of the Q‐factors discrete distribution.

Here, we demonstrate mode‐locking in surface emitting random THz QCLs. This is achieved by altering the intracavity field of an electrically pumped semiconductor heterostructure 2d laser resonator,^[^ ^32^ ^]^ varying the reflectivity at its top surface, by using two different configurations. In the first case (external graphene, EG, configuration), we couple on‐chip an ultrafast (∼ps) GSA mirror^[^ ^33^ ^]^ on the top surface of a random resonator (**Figure** **1**a). In the second case (integrated graphene, IG, configuration), we intracavity embed a seven layer graphene (7LG) film grown by chemical vapor deposition (CVD), in the holes of a set of randomly arranged scatterers acting as light outcouplers (Figure 1b). Self‐mixing intermode beatnote (SMIB) spectroscopy is then employed to identify the phase locked random modes. This is the first experimental report of a mode locked random resonator in the THz range, in which continuous, phase‐locked, inherently space‐incoherent, random modes are generated and downconverted in the RF domain without leading to the emission of short pulses.

Graphene has been widely exploited as SA in the infrared.^[^ ^37^, ^38^, ^39^, ^40^, ^41^, ^42^, ^43^ ^]^ Mode‐locked lasers using GSAs have been demonstrated from ≈800 nm^[^ ^35^ ^]^ to ≈970 nm,^[^ ^39^ ^]^ 1.1 µm,^[^ ^45^ ^]^ 1.5 µm,^[^ ^36^ ^]^ 2 µm,^[^ ^37^ ^]^ 2.4 µm,^[^ ^38^ ^]^ and 2.8 µm.^[^ ^39^ ^]^ While single and multiwall nanotubes have been used to mode lock lasers in the in the visible/near infrared^[^ ^40^, ^41^ ^]^ their typical bandgaps mean they are not ideally suited to mode lock in the THz region, corresponding to 10–14 meV.

The gapless graphene nature allows broadband operation.^[^ ^42^ ^]^ The ultrafast recovery time,^[^ ^43^, ^44^ ^]^ low (∼3 W cm^−2^ in the THz^[^ ^33^, ^45^ ^]^, and ∼300 MW cm^−2^ in the NIR region^[^ ^34^ ^]^) saturation fluence, and ease of fabrication,^[^ ^46^ ^]^ make graphene an ideal SA also in the THz,^[^ ^33^ ^]^ exploiting integrated architectures where intracavity power intensities of the order of tens of W mm^−2^ are available.

By coupling a GSA reflector with a QCL frequency comb,^[^ ^45^ ^]^ phase locking of optical modes, in the operational laser regime in which this cannot spontaneously occur due to the chromatic dispersion,^[^ ^47^, ^48^ ^]^ was demonstrated. The combination of fast gain saturation provided by the GSA, and Fresnel reflection at the graphene surface, forced the QCL into a frequency modulation (FM) comb state, without the emission of short pulses.^[^ ^45^ ^]^ Theoretical investigations on Fabry‐Perot (FP) QCLs combs also highlighted the key role of large cavity mirror losses^[^ ^49^, ^50^ ^]^ in promoting the proliferation of phase‐locked modes over the whole gain bandwidth, through the alteration of the intracavity field.

Our rationale here is to explore the RL intermodal correlations sensitivity to the reflectivity changes of the laser outcoupling elements, mimicking the physical phenomena arising in coherent feedback mode‐locked RLs,^[^ ^26^ ^]^ or in the mentioned broadband frequency‐modulated combs.^[^ ^50^ ^]^

### 1.1 Device Design and Simulations

The random resonators are fabricated as follows: a 2d random arrangement of circular scatterers is lithographically patterned on the top resonator surface as for Ref. [23], and etched across the top highly doped contact. This enables simultaneous control of optical feedback and extraction mechanisms. THz photons undergo multiple elastic scattering events and are confined and amplified inside the active material due to the high refractive‐index contrast between Au‐coated semiconductor and air holes. When the photon in‐plane momentum is reduced to zero, light is extracted vertically through the holes and coupled into free space. The holes scatterers and their distributions (filling factor, FF) affect slope efficiency, far field profile, spectral coverage, and maximum current density, as detailed in Ref. [23]. The double metal random resonator is processed with an irregularly shaped border partially coated with a 10 nm‐thick lossy Cr layer to suppress periodic whispering gallery and FP modes (see the Experimental Section). In the IG configuration, a CVD 7LG is then transferred onto the open holes (see the Experimental Section). A set of resonators having FF in the range ∼8%–26% is fabricated and tested (see the Supporting Information). While the threshold current density is mostly independent from FF, the overall laser performances improve significantly increasing FF (see the Supporting Information), as expected from the increase of randomness in resonators^[^ ^30^ ^]^ with a higher density of holes. The effect of graphene integration on QCL optical performances is investigated in the Supporting Information. An optimal filling factor ≈22% (see Figure S17, Supporting Information) is found to be the best compromise between higher scattering strength in highly disordered media and loss increase in high hole density random photonic structures.

We then simulate the optical modes quality factors, *Q*_tot_, and photon loss rates (see the Supporting Information) of the 2d disordered resonator in 3 distinctive configurations: i) standard RL^[^ ^23^ ^]^ (pristine); ii) the same RL coupled with an external solution‐processed MLG (EG) mirror in close proximity (50 µm) of its top surface; and iii) RL with a random surface pattern of holes filled with CVD graphene (IG). The optical parameters are described in the Supporting Information.

The external mirror configuration decreases the photon loss rate in the 2.8–3.1 THz range (Figure 1c), corresponding to the lower half of the QCL gain bandwidth.^[^ ^52^ ^]^ This promotes a larger number of discrete modes to reach the lasing threshold, thus enriching multimodal emission and enhancing the probability of cross‐mode interaction. The frequency dependence of the IG photon loss rate is instead comparable with the pristine case, even if the values are slightly higher, aside from discrete spectral intervals where the opposite occurs (Figure 1c). The RL modes compete for the available gain both in the spectral and spatial domains as an effect of spatial hole burning.^[^ ^22^ ^]^ Then, a set of discrete modes having the lowest losses and the highest Q‐factors are selected through optical feedback. For the 2d random resonators, a standard Gaussian‐like distribution of the Q‐values is found (Figure 1d), with the theoretical Q‐factors of the resonator modes in Figure 1e spreading evenly around a mean *Q*_tot_ = 37 for the pristine case, 36 for the EG mirror, and slightly lower (30) for the IG configurations. The width of the *Q*_tot_ distribution is consistently narrower when graphene is employed, especially for IG. The same behavior is found for all investigated photonics patterns, resonator dimensions and FFs (see the Supporting Information), implying this is as a universal property of IG resonators. A narrower *Q*_tot_ distribution implies a higher mode competition, possibly eliciting a stronger mode proliferation. On the other hand, the role of the photon losses on cross‐mode correlations could be more complex. The increase in photon losses is a direct result of the insertion of an absorbing surface at the light outcoupler holes. Being graphene part of the resonator, it inherently affects the feedback in a similar way of increased mirror losses in a FP laser. Such a condition favors the cross‐steepening, i.e. the mechanism responsible for the transition from an uncorrelated multimode regime to frequency modulated QCL combs.^[^ ^49^, ^50^ ^]^ The integration of MLG onto the random scatterer leads to a strong increase of the scatterers intracavity reflectivity (see the Supporting Information). For the IG architecture it is mostly the linear optical response of the MLG to play a role,^[^ ^53^ ^]^ SA in the graphene reflector may additionally contribute to regularize the phase coherence of the locked modes.^[^ ^45^ ^]^

### 1.2 Transport, Optical, and Intermodal Beatnote Experimental Analysis

**Figure 2:**
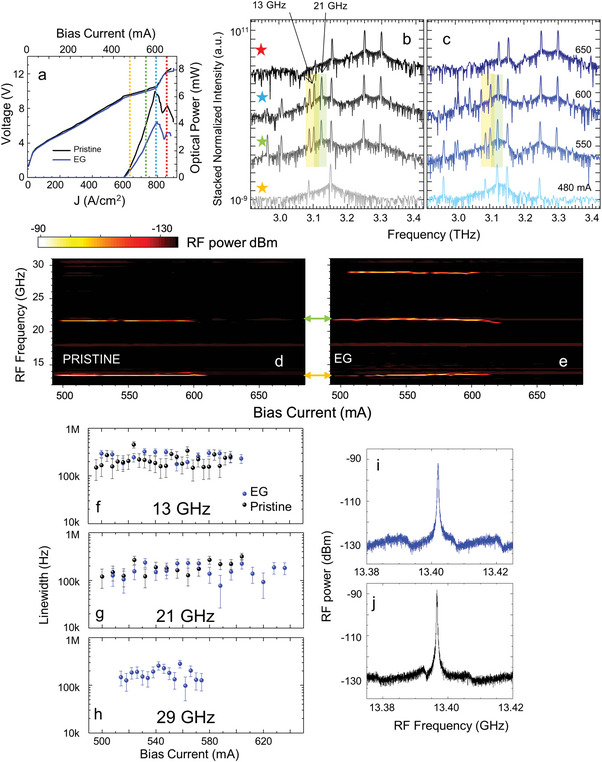
Intermode beatnote in graphene external cavity mirror. a) Current density–voltage (*J*–*V*) and current density–optical power (*J*–*L*) characteristics of the RL of Figure 1a, measured at a heat‐sink temperature of 18 K, while driving the lasers in quasi‐CW mode with a pulse width ∼ 5 µs and a repetition rate ∼100 kHz (50% duty cycle) in vacuum, for the bare laser resonator (pristine) (black) and EG (blue) configurations. b,c) FTIR stacked spectral emission of the RL‐QCL of Figure 1a in pristine and EG configurations, at the 4 driving currents indicated as vertical lines in panel (a): from top to bottom, 650 mA (red), 600 mA (light blue), 550 mA (green), and 500 mA (yellow). The star symbols in (c) indicate the driving currents, with the same color code. The yellow and green regions in (b,c) mark the spectral lines responsible for the 13 and 21 GHz beatnotes, respectively. d,e) Intermode beatnote maps measured on the bare resonator and in the EG configuration. The beatnote signal is extracted from the bias line with a bias‐tee and recorded with an RF spectrum analyzer (RBW: 5 kHz, video bandwidth (VBW): 5 kHz, sweep time (SWT): 20 ms, RMS acquisition mode). All measurements in (b–e) are performed in CW at a fixed heat sink temperature of 18 K. f–h) Intermode beatnote linewidths as a function of driving current for the ∼13, 21, and 29 GHz beatnotes, on the bare random QCL resonator (black) and in the EG (blue) random QCL. i,j) Intermode beatnote traces measured at *I* = 580 mA for the ∼13 GHz beatnote in the bare (blue) and EG (black) random resonator.

To corroborate these assumptions, we perform a comparative study of the spectral emission in different regimes along the light–current density (*L*–*J*) characteristic (**Figure** **2**a) in the pristine resonator and in the EG architecture. The results are confirmed by analogous experiments on several random QCLs having dissimilar resonator size and FF (see the Supporting Information), and emitted power >3 mW. EG is not affecting the overall laser dynamic range and lasing threshold, aside from an ≈40% power attenuation, compatible with the reflection losses induced by the EG mirror SiO_2_/Si substrate and the saturated and unsaturated absorptions form the GSA surface^[^ ^33^ ^]^ (see the Experimental Section).

We then extract the experimental quality factor from the relation^[^ ^54^ ^]^ *Q*_exp_ = 2*πn*_eff_/*λg*Γ*J*_th_, where *n*_eff_ = 3.66 is the active region effective refractive index, *λ* is the wavelength, Γ = 1 is the confinement factor of the double‐metal resonator, *g* = 0.061 cm A^−1^ is the active medium gain,^[^ ^52^ ^]^ and *J*_th_ = 590 A cm^−2^ is the experimental threshold current density. Since the presence of the graphene mirror does not affect *J*_th_, by averaging over the laser spectral range, we estimate *Q*_exp_ = 66 for both the EG and the pristine cases, higher than the 3d simulation *Q*_tot_, leading to total extraction losses *γ*_tot_ = *ν* × *n*_eff_/(*Q*_tot_ × *c*) = 10.8 cm^−1^ and *γ*_tot_ = 10.7 cm^−1^ for EG and pristine cases, respectively, where *c* is the light speed and *ν* the random mode frequency. The calculated average photon loss rates due to surface emission (see the Supporting Information) *γ*_rad_ = 2.1 cm^−1^ (pristine) and *γ*_rad_ = 2.2 cm^−1^ (EG) suggest that the main loss channel is not radiative. We attribute the discrepancy between *Q*_exp_ and calculated *Q*_tot_ to an underestimation of the non‐radiative Ohmic losses, whereas both simulations and experiments consistently confirm the negligible impact of EG on Q‐factors.

The Fourier transform infrared (FTIR) emission spectra measured in CW (Figure 2b,c) and in the two configurations show differences. For EG (Figure 2c), we observe a richer multimodal behavior with a larger number of spectral lines. Slightly above threshold, the EG QCL emits 3 modes centered at ∼2.96, 3.13, and 3.25 THz, not visible in the emission spectra of the bare random resonator, i.e. when the graphene mirror is absent (Figure 2b). At higher currents, a group of 3 spectral lines in the 3–3.1 THz range and one isolated and weaker spectral line at 3.21 THz appear only when the resonator is coupled with the graphene mirror (EG). The presence of these sets of random lines in the lower half of the spectral bandwidth can be ascribed to the decreased photon loss rate expected in the EG configuration (Figure 1c), and is also an indication of random lasing with incoherent feedback in specific spectral windows.

We then monitor the intermodal beatnote to reveal the cross‐mode correlation effects. The beatnote maps (Figure 2d,e) show differences, in agreement with Figure 2b,c. In both EG coupled and bare random resonators, we unveil 2 single and narrow beatnotes centered at 13 and 21 GHz, respectively, with the 21 GHz beatnote detected for a wider bias range (>20 mA driving current) in EG. A third single and narrow beatnote at 29 GHz is visible only in the EG case (Figure 2e). In our 2d random design, multimodal emission does not comprise a set of discrete periodically spaced modes. Thus, instead of the appearance of one single and narrow intermode beatnote at a specific RF frequency, corresponding to the mode spacing, multiple beatnotes are expected to appear, whose frequencies are related to the spectral spacing between cross‐correlated lasing lines.

Numerical simulations reveal that our resonators sustain a variety of spectrally close (or degenerate) random modes with very similar Q‐factors and extraction losses, competing in the whole laser gain bandwidth, meaning that the measured beatnotes may arise from a complex locking process involving a large number of spectrally close random modes.

We assign the 13 GHz beatnote to the beating of the two modes highlighted by the yellow area in Figure 2b,c (≈3.089 and 3.102 THz), and the 21 GHz beatnote to the beating of the two modes highlighted by the green area in Figure 2b,c (≈3.130 and 3.102 THz). The beatnote at 29 GHz, retrieved only in the EG configuration, is compatible with the beating of at least 4 random modes (∼3.249 and 3.220 THz, the latter absent in the bare resonator, and ∼3.130 and 3.159 THz). In this case, the beating is a direct effect of the presence of graphene. Figure 2f‐h plot the beatnote linewidths (LWs) for the 3 beatnotes, extracted from the Lorentzian fit of the beatnote acquisitions, whose prototypical traces are in Figure 2i,j. LWs in the range 150–250 kHz are found in both configurations and for all beatnotes, i.e. a factor ∼30–50 higher than the typical free‐running linewidth in FP frequency‐combs with the same active region design,^[^ ^52^ ^]^ but ∼3 orders of magnitude smaller than when phase coherence is lost,^[^ ^45^, ^55^ ^]^ signature that a more limited number of modes is here phase‐locked.^[^ ^56^ ^]^

**Figure 3:**
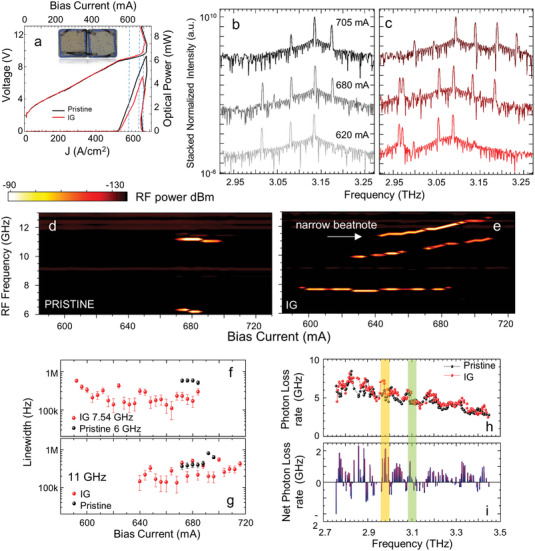
Cross‐mode correlation in graphene integrated RLs. a) Comparison of *J*–*V* and *J*–*L* of the two random QCLs shown in the inset, having identical geometrical design, *r*/*a* = 10% (filling fraction ∼ 3%), total area ∼ 0.105 mm^2^, from a 2d arrangement of 41 holes with 5 µm radius, distributed on a square area with *L* = 325 µm, with IG inside the scattering holes on one of the two devices. Measurements are performed at a heat‐sink temperature of 18 K, while driving the lasers in quasi‐CW mode with a pulse width ∼ 5 µs and a repetition rate ∼ 100 kHz (50% duty cycle) in vacuum. Inset: Optical images of the two random QCLs, with the IG device on the left. For accurate comparison, the two lasers are realized within the same fabrication run, mounted on the same chip, and tested during the same cooling cycles. b,c) FTIR stacked spectral emission of pristine and IG R‐QCLs, at the 3 driving currents indicated by the dashed vertical lines in (a): from top to bottom, 705, 680, and 620 mA. d,e) Intermode beatnote maps measured for the pristine and IG device. The beatnote signal is extracted from the bias line with a bias‐tee and recorded with an RF spectrum analyzer (RBW: 5 kHz, video bandwidth (VBW): 5 kHz, sweep time (SWT): 20 ms, RMS acquisition mode). All measurements in (b–e) are performed in CW, at a fixed heat sink temperature = 18 K. Intermode beatnote linewidths as a function of driving current for f) ∼6 GHz (black dots) and ∼7.54 GHz (red dots) and g) ∼11 GHz beatnotes, on pristine (black) and IG (red) random resonators. The ∼11 GHz beatnote in the IG configuration is the narrower of the two in (e), highlighted by the white arrow. h) Computed photon loss rates in pristine (black) and IG (red) architecture. i) Net photon loss rate, calculated as the difference between red and black curves in (h), after interpolation of loss coefficients versus the simulation eigenmodes. The yellow and green shadowed regions in (h) and (i) highlight the spectral ranges where the strongest experimental mode proliferation is observed.

The EG introduction does not have any significant impact on the LW of the 13 and 21 GHz beatnote, meaning that the beating process likely arise from only 2 modes.The presence of the external GSA mirror induces an intensity dependent optical feedback in the RL that results in a richer multimodal emission and an intensity dependent optical feedback in the RL. The computation methods used to extract *Q*_tot_ and the photon loss rates do not account for a possible adaptive optical response, since they only capture the electromagnetic linear response in passive systems.

We then investigated a set of IG random lasers (see Experimental Section). First, we compare the *L–J* (**Figure** **3**a), the CW emission (Figures 3b,c) and the intermode beatnote maps (Figures 3d,e) for IG and pristine (bare random resonator with no graphene in the holes) devices, simultaneously implemented on the same chip. *Q*_exp_ are comparable since the threshold is not affected by graphene integration. We extract *Q*_exp_ = 82 for IG and *Q*_exp_ = 83 for pristine RL, more than twice the simulated values (36 and 37, respectively).

A richer spectral emission is retrieved in the IG device. At 680 mA, the single mode at 2.96 THz, of low intensity, retrieved in the bare resonator, is substituted by a much intense couple of random modes in the IG device, having frequency spacing ∼8 GHz.

The comparison between the intermodal beatnote map of the bare RL (Figure 3d) and of the IG (Figure 3e) reveals major differences.

**Figure 4:**
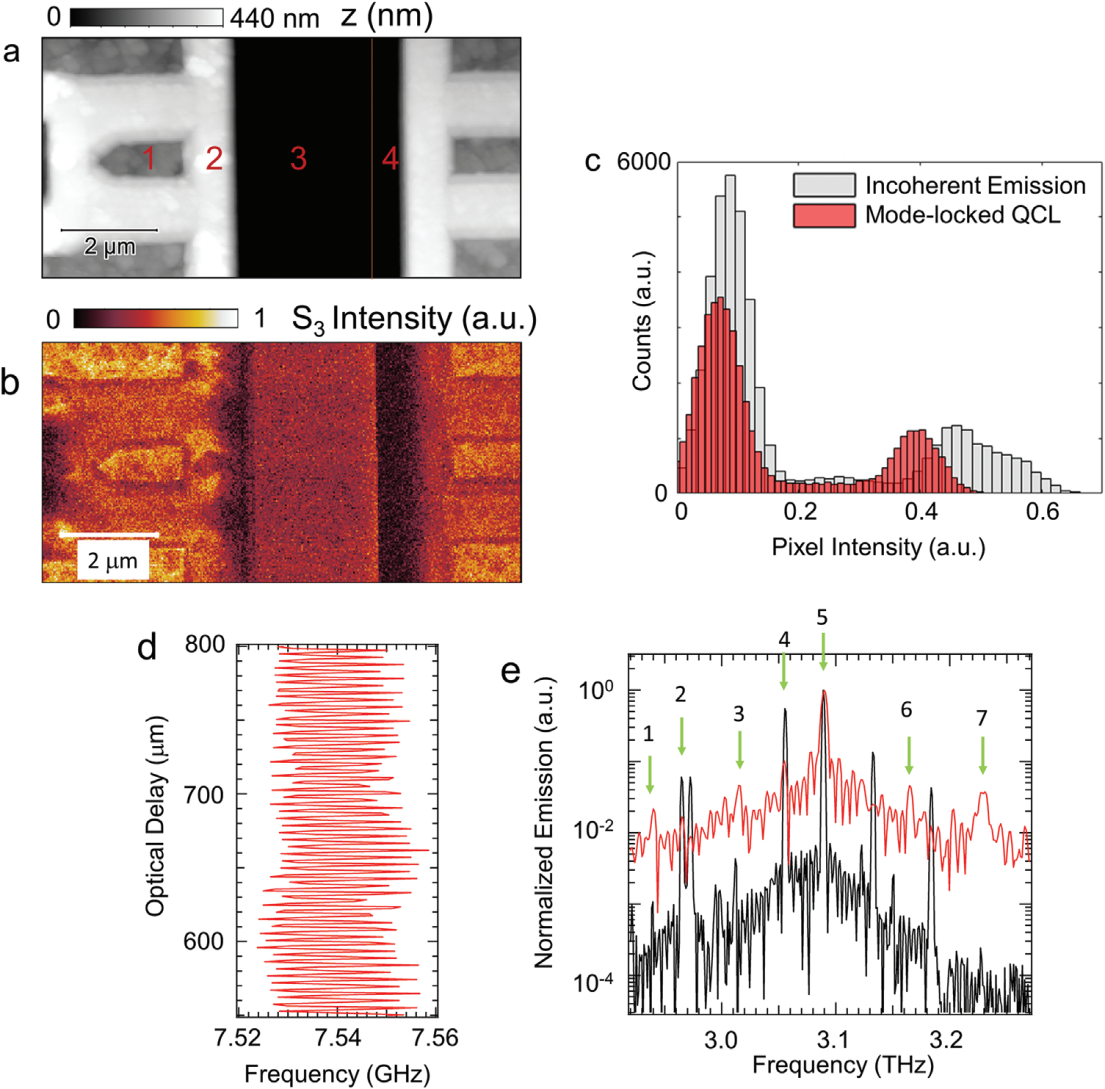
Hyperspectral near‐field imaging in mode‐locked regime. a) Sample topography (*z*) and b) self‐mixing signal intensity *s*_3_ demodulated at the third harmonic of the tapping frequency *ω*_t_, collected with a speed of 2 µm s^−1^ corresponding to 6 ms pixel^−1^, acquired on a Si sample having inhomogeneous doping, distributed as follows: region 1 is implanted p‐type (4 × 10^19^ cm^−3^), region 2 and 4 are implanted n‐type (1 × 10^17^ cm^−3^), region 3 is the homogeneously p‐doped substrate (2 × 10^16^ cm^−3^). The SM signal is acquired driving the IG Random QCL of Figure 3, in CW, at 620 mA and *T* = 22 K, corresponding to the mode‐locked regime. c) Comparison of pixel intensity distributions of near‐field images acquired with the IG random QCL of Figure 3 in CW at 620 mA, corresponding to the mode‐locked regime (red), and biased at 590 mA (gray), corresponding to the incoherent emission regime. d) Central frequency of ≈7.54 GHz beatnote on the beatnote map of the IG Random QCL of Figure 3e, recorded as a function of the optical path variation with steps of 8 µm, here shown for a 300 µm long section of the full scan. e) Comparison of normalized emission spectra, acquired via FTIR (black line) and by Fourier transforming the full beatnote interferogram trace in (d) (red line). The green arrows highlight the phase‐locked modes. The spectral lines extracted upon FT filtering of the SMIB trace are compatible with the observed beatnote at 7 GHz, owing to the beating of the two sets of modes at 2.97 THz, also present in the red curve, although with relative lower intensities. They can also arise from downconversion occurring in the laser resonator, e.g., the 3 modes labeled 5, 6, 7 at 3.091, 3.163, and 3.232 THz can lead to a frequency difference mixing at the ∼7.54 GHz experimental beatnote, similarly to lines 3, 4, and 5 (∼3.012, 3.055, and 3.091 THz).

In particular, the beatnote ≈7.54 GHz and the double beatnotes tuning between 10 and 11 GHz and 11 and 12 GHz (Figure 3e), respectively, retrieved in the IG laser, have a different origin with respect to the ≈6 GHz and 11 GHz single beatnotes, measured, always at a fixed frequency, in the bare RL, in the driving current range 670–700 mA (Figure 3d). In IG, we typically observe a stationary frequency modulation of beatnotes, owing to a frequency modulation of the beating modes. Such behavior is reflected in the broadening of the RF beatnote or, as in the present case, in the appearance of broader (width ≥1 MHz) replica in the beatnote maps. This is particularly true when random modes with weak and/or uneven intensity, varying over a 50 dB range, are involved in the locking process, as in the present case (Figure 3c). This supports the idea that the individual beatnotes, retrieved in the IG, arise from a complex nonlinear mixing between several correlated modes over the entire emission band, as confirmed by the SMIB spectroscopy experiments described in the paragraph below (see **Figure** **4**).

Figures 3f,g show the corresponding beatnotes linewidths retrieved in the IG and bare resonator. The IG beatnotes (Figure 3f) tuning ∼ 7.54 and 11 GHz, are a factor of two and three times narrower, respectively, than the beatnotes at 6 and 11 GHz, measured in the corresponding RL without graphene (Figure 3g), hence suggesting a strengthening of the cross‐mode locking for the modes involved. The calculated photon loss rates (Figure 3h) show an increase in the IG case, with the net photon loss rate (Figure 3i) calculated as the difference between IG and pristine, consistently above zero in the frequency regions of the beating lasing modes. We estimate an ∼6.6 GHz average loss rate for IG, i.e., 30% higher than the pristine loss rate (≈5.2 GHz) for the locked modes around 2.95 THz, and ∼4.5 GHz average IG loss rate, 15% higher than the pristine loss rate (∼3.8 GHz) for the beating modes at ≈3.12 THz. The presence of narrower and more persistent beatnotes across a wider operational range of the RL in IG devices, corresponding to random modes with higher photon losses, is consistently found in a set of eight random QCLs, having different FF, under the same experimental conditions (see the Supporting Information). This scenario resemblesthose reported in Refs. [38, 39]. In the IG random laser, the mode intensities vary continuously upon subsequent multiple scattering, since the scatterer losses are shaped by the optical absorption of the intracavity graphene reflector. This results in a complex interference phenomenon among the neighboring random modes, whereby partial frequency synchronization could arise in analogy to nonlinear optical phenomena, such as the antiphase synchronization in coupled clocks.^[^ ^57^ ^]^ Although in all cases the optical intensity on the GSA is above the saturation threshold,^[^ ^33^, ^58^ ^]^ all the main laser figures of merit, including the threshold and power (see the Supporting Information and Figures 2 and 3), are negligibly affected by the integration of the GSA in each investigated random resonator.

### 1.3 Near‐Field Nanoscopy

We then compare the near‐field response of the IG random‐QCL of Figure 3 in the incoherent emission regime at 590 mA, and in the mode‐locked regime, i.e., when a single beatnote is detected, i.e., at 620 mA, by scattering‐type scanning near‐field optical microscopy (s‐SNOM)^[^ ^59^ ^]^ (see the Supporting Information). We map a Si static random access memory (SRAM) test sample from Bruker (Figure 4a) with subdiffraction spatial resolution and retrieve, by lock‐in detection of the self‐mixing signal at harmonics of the tip tapping frequency, the near‐field scattering in the 2.9–3.3 THz range, where conventional table‐top systems capable of hyperspectral imaging, like TDS s‐SNOM, cannot operate, since they show a >2 orders of magnitude drop of their signal‐to‐noise ratio at spectral frequency > 1.5 THz.^[^ ^60^ ^]^ The third harmonics near‐field signal, for the mode‐locked laser, is displayed in Figure 4b, while harmonics up to the fifth are reported in the Supporting Information. The narrowing of the single pixel intensity distribution in Figure 4c demonstrates a significant improvement of the speckle density^[^ ^5^ ^]^ in the mode‐locking regime compared to standard multimode emission.^[^ ^5^ ^]^

To corroborate the evidence of mode locking, we exploit the sensitivity of the beatnote to the feedback in SMIB spectroscopy experiments to determine the mode‐locked modes.^[^ ^61^, ^62^ ^]^ While intermode beatnote maps can only provide a preliminary indication of mode‐locking, SMIB is an experimental technique commonly adopted, in conjunction with the beatnote analysis, to demonstrate and assess, unambiguously, phase‐locking in QCLs, during their operation as frequency combs.^[^ ^63^, ^64^ ^]^

We measure the beatnote frequency shift as a function of delay (Figure 4d), when the QCL emission is sent to an Au mirror, through a delay line. Since the beatnote is sensitive only to the mode‐locked modes which, due to the spatial incoherence of random QCLs, are spectrally isolated (Figure 4e), the beatnote shift can be used to retrieve the sample response at the mode‐locked THz frequencies.^[^ ^65^ ^]^ Beatnotes, generated by s of mode‐locked modes, could then allow one to perform fast hyperspectral THz imaging, removing the need to acquire the whole spectrum for each pixel, enabling an extremely fast acquisition scale (hundred µs/pixel).

We exploit the sensitivity of the SMIB signal only to the phase correlated modes,^[^ ^66^ ^]^ as a further proof of mode‐locking. The spectral content of the SMIB trace, obtained via Fourier transform (Figure 4e), unequivocally identifies the locked modes. There are at least seven sets of modes in the SMIB curve of Figure 4e, meaning that all modes retrieved in the spectra of Figure 3c are locked in‐phase. Furthermore, when optical feedback is introduced, we observe an ∼2 MHz shift in the beatnote frequency, 10 dB variation of beatnote power and a reduction of a factor ∼5 of the beatnote linewidth (see the Supporting Information). This is a further proof of mode‐locking, as a modulation of the optical feedback can only affect the phase of the locked modes involved in the beatnote formation,^[^ ^66^ ^]^ leading to a variation of the beatnote linewidth and self‐mixing signal‐to‐noise ratio.

Note that a different surface graphene hole pattern may lead to phase locking of couples of individual random modes without necessarily implying that the random laser is fully mode locked, as in the present case (see the Supporting Information).

Conversely, for unlocked modes, whose beating is reflected in a broad beatnote, a noiseless SMIB trace, comprising specific frequency/linewidth variations of the beatnote, cannot be retrieved (see Figure S21, Supporting Information).

As a further and independent proof of phase coherence we fully frequency stabilize the IG random QCL of Figures 3 and 4, upon injection of a RF signal at the same frequency of the narrow beatnote, in an all‐electric injection locking experiment (see Figure S22, Supporting Information), proving that the intermode BN power is almost completely locked.

## 2 Conclusions 

The existence of nonlinear interactions and mode‐locking among random modes in miniaturized electrically pumped random QCLs paves the way for the development of a novel class of miniaturized, chip‐scalable, electrical devices with tunable optical properties, opening the path for multispectral imaging, free form speckles and spatial cross talks, to be used in biomedicine, cultural heritage and security, and for multifrequency high‐precision spectroscopy, stemming from the mode‐locked nature of the random emission. This can be also used as a novel platform for reservoir computing in the far‐infrared and adds a fascinating achievement in the physics of complex systems.

## 3 Experimental Section

### Electromagnetic Simulations of Random Resonators

The 3d model implemented in Comsol Multiphysics, featuring finite element analysis in the frequency domain, comprises a double‐metal resonator, with the top and bottom cladding layer treated as perfect electric conductors. The 10‐µm‐thick active medium is GaAs with isotropic refractive index *n*_GaAs_ = 3.665. To provide smooth boundary conditions for the resonator modes, the mesa border of the photonic structure is irregularly shaped with protrusions ≈ 25 µm, i.e. comparable with the GaAs waveguide mode wavelength, and coated with a transition material having a complex refractive index *n*_Cr_ = 4.43 + i0.31, accounting for the 10 nm Cr layer. The resonator is covered by a 100‐µm‐thick air volume, and surrounded by scattering conditions on all the boundary surfaces to mimic free‐space propagation. In the EG configuration, an undoped Si element is placed 50 µm from the resonator top emitting surface. The Si/air interface facing the random holes is then modeled as a transition boundary condition with the graphene optical constant *n,k* and effective thickness 50 nm. Similarly, in the IG configuration, individual hole/air interfaces are transition surfaces with the graphene *n,k* and effective thickness 2.4 nm (see Supporting Information).

### Random QCLs Fabrication Procedures

The 11‐µm‐thick AlGaAs active regions are grown via molecular beam epitaxy (MBE) and feature a 3‐quantum well resonant phonon architecture.^[^ ^67^ ^]^ The layer sequence is **5.5**/11.0/**1.8**/11.5/**3.8**/9.4/**4.2**/18.4 (in nm), where Al_0.15_Ga_0.85_As layers are shown in boldface, and the underlined number indicates the 2 × 10^16^ cm^−3^ Si‐doped layer, terminated by a 700‐nm‐thick highly doped (2 × 10^18^ cm^−3^) GaAs cladding. For the double‐metal structure formation, Au—Au thermo‐compressive bonding of the QCL wafer is performed onto an n+‐GaAs carrier wafer, followed by the removal of the MBE carrier GaAs substrate and the Al_0.5_Ga_0.5_As etch‐stop layer. By using a laser writer, the Au top contact with the air holes is then defined, by using a pattern design implemented with the help of a MATLAB script for 2d random number generation.^[^ ^23^ ^]^ To ensure the light outcoupling, the doped GaAs top layer underneath the holes is totally removed, by means of an inductive plasma reactive‐ion etching (ICP‐RIE) process. The absorbing boundary conditions are then realized by lithographic definition of a 10‐nm‐thin, 25 µm‐wide Cr frame with irregular protrusions of average size ≈25 µm. Then, the mesa is etched using a second ICP‐RIE process, for optimal vertical sidewalls. For the IG devices, a set of resonators having size *L* = 325 µm and filling factors ranging from 8% to 26% is iterated at least twice on the sample surface, to realize a couple of identical graphene‐coated and uncoated random resonators. Then, CVD MLG is transferred onto the top contact. To ensure maximum adhesion, MLG is realized by stacking sequentially SLGs grown on Cu (Graphenea, Inc.) using polymer‐assisted Cu wet‐etching,^[^ ^68^ ^]^ until the desired stack thickness is achieved (7 layers). Each step of the transfer comprises spin‐coating of 270 nm PMMA on the graphene/Cu sample, wet‐etching removal of Cu with 0.5 m ammonium persulfate solution, and transfer of PMMA/graphene on the next‐step substrate. To minimize the interlayer contaminants from the wet etching, the entire stack is realized by using the Cu epitaxy substrate as sacrificial carrier, then transferring it on the random‐QCL top contact as final step. After polymer removal, MLG is lithographically etched from half of the lasers with an O_2_‐assited RIE. To remove the polymer residues, the sample is cleaned with a 12 h acetone soak and fast annealing at 320 °C for 10min. The resulting RLs, tested after one year, show complete stability, with no change in the optical and electrical performances.

### External Graphene Mirror Preparation

For the EG experiment, a graphene reflector prepared by liquid phase exfoliation of graphite in a water/surfactant solution is used,^[^ ^33^ ^]^ assembled by ultrasonicating graphite flakes in deionized water with sodium deoxycholate, then vacuum filtering the solution with a 100 nm pore‐size nitrocellulose mesh.^[^ ^33^ ^]^ The ink is then placed on the 420µm‐thick intrinsic high‐resistivity Si/SiO_2_ double polished wafer (acting as a back reflection mirror), annealed at ≈80 °C for 2 h, and cleaned. The film is 50 nm thick, and covers a surface ∼1 cm^2^. The resultant mirror (∼42% transmissivity in the THz) behaves as a THz SA, providing 80% transparency modulation, as a result of intraband induced absorption bleaching.^[^ ^33^ ^]^

### Transport and Optical Measurements

Individual dyes each containing 1 IG/pristine device couple are in‐soldered onto a Cu bar and wire bonded regularly along the random resonator perimeter to ensure uniform current distribution, then mounted on the cold‐head of a liquid‐He cryostat with THz transparent optical windows. For the EG experiment, the graphene reflector is closely coupled to the emitters holes, by placing the mirror ∼ 0.05–0.1 mm away from the top surface, covering the entire RL surface.

### Nonlinear Absorption Parameters

For the ink‐based GSA sample used in the EG configuration, a modulation depth *α*_s_ ≈ 80% is found experimentally^[^ ^33^ ^]^ with a saturation intensity ≈ 6.7 ± 1.0 W cm^−2^. In the CVD film, *α*_s_ ≈ 18%^[^ ^58^ ^]^ for 7LG with a saturation intensity ≈1.8 W cm^−2^. An ultrafast recovery time ≈2–3 ps^[^ ^43^, ^44^, ^69^ ^]^ is measured at THz frequencies.

### SMIB Spectroscopy

The IG random‐QCL radiation was collected by a parabolic mirror and sent through a delay line to an Au mirror. The reflected radiation was then coupled back into the QCL cavity via the same path. The feedback phase can be changed by varying the delay line position. The THz modes spacing changes due to the feedback because of the Lang–Kobayashi shift^[^ ^65^ ^]^ of the single modes, resulting in a variation of the beatnote frequency at each step of the delay line (Figure 4d). The fast Fourier transform of the resulting time trace reveals the mode‐locked modes generating each beatnote, whereas the spectral amplitude is a measure of the beatnote shift sensitivity to each THz frequency. This allows using the beatnote shift to monitor the feedback intensity, i.e., the sample reflectivity, directly at the mode‐locked THz frequencies without the need of performing a few hours long time scan for each pixel.

## Conflict of Interest

The authors declare no conflict of interest.

## Author Contributions

M.S.V. conceived the concept. A.D.G. fabricated the random lasers, integrated the devices with graphene, set up the transport and optical experiment, and acquired the experimental data. A.D.G. and V.P. performed numerical simulations and interpreted the data. E.R. provided technical support in the graphene transfer and in the QCL fabrication steps. E.A.A.P. and V.P. performed and analyzed the self‐mixing imaging experiment. H.E.B., D.A.R. L.L., A.G.D., and E.H.L. grew by molecular beam epitaxy the QCL structure. A.C.F. provided and characterized the external graphene mirror. The manuscript was written by A.D.G., A.C.F, and M.S.V. M.S.V. coordinated and supervised the project.

## Code Availability

The codes and simulation files that support the plots and data analysis within this paper are available from the corresponding author upon reasonable request.

## Supporting information

Supporting Information

## Data Availability

The data that support the findings of this study are available from the corresponding author upon reasonable request.
