# Supplementary material for: Self‐Induced Mode‐Locking in Electrically Pumped Far‐Infrared Random Lasers
Source: Adv Sci (Weinh). 2023 Jan 27;10(9):2206824. doi: 10.1002/advs.202206824 (PMC10037977; doi:10.1002/advs.202206824)
Supplement: Supplementary file 1 — Supporting Information [file ADVS-10-2206824-s001.pdf]

ADVANCED SCIENCE
Open Access

Supporting Information

for *Adv. Sci.*, DOI 10.1002/advs.202206824

Self-Induced Mode-Locking in Electrically Pumped Far-Infrared Random Lasers

*Alessandra Di Gaspare, Valentino Pistore, Elisa Riccardi, Eva A. A. Pogna, Harvey E. Beere, David A. Ritchie, Lianhe Li, Alexander Giles Davies, Edmund H. Linfield, Andrea C. Ferrari and Miriam S. Vitiello**

Self-induced mode-locking in electrically-pumped far-infrared random lasers

Alessandra Di Gaspare,[1] Valentino Pistore,[1] Elisa Riccardi[1], Eva A. A. Pogna[1], Harvey E. Beere[2], David, A. Ritchie,[2] Lianhe Li,[3] A. Giles Davies[3] Edmund H. Linfield,[3] Andrea C. Ferrari and Miriam S. Vitiello[1]

[1]*NEST, CNR - Istituto Nanoscienze and Scuola Normale Superiore, Piazza San Silvestro 12, 56127, Pisa, Italy*
[2]*Cavendish Laboratory, University of Cambridge, Cambridge CB3 0HE, UK*
[3] *School of Electronic and Electrical Engineering, University of Leeds, Leeds LS2 9JT, UK*
[4]*Cambridge Graphene Centre, University of Cambridge, Cambridge CB3 0FA, UK*

I. Numerical analysis of random resonators

We engineer the random photonic resonators through 3d EM simulations, employing a finite element procedure (COMSOL Multiphysics). We extrapolate the total 3d quality factors, Q_{tot}, as a direct output of the simulations. The total resonator losses, in Hz, are defined as $\gamma_{tot} = \frac{\nu}{Q_{tot}}$, where ν is the eigenmode frequency of a specific random mode. The photon loss rate, γ_{rad}[1], is calculated as the ratio between the total power output, retrieved by integrating the EM outflow power over the top surface, and the total internal electric field energy, upon integration of the EM energy over the resonator volume. The loss terms can be alternatively expressed in cm^{-1}, by dividing the loss rate [Hz] by the group velocity $v_g \sim c/n_{eff}$ with $c = 3 \times 10^8$ m/s the speed of light and $n_{eff} = 3.665$ the GaAs effective refractive index. The ohmic losses are then retrieved as the difference between the total γ_{tot} and the radiative term γ_{rad}.

II. Graphene optical constants in 3d EM simulations

The optical response of single layer graphene, SLG, is governed by both intraband and interband transitions,[1] whose frequency dependent relative contributions may differ across the EM spectrum. Interband transitions dominate at visible and infrared wavelengths, while intraband transitions are usually dominant at THz frequencies.[2,3,4] The total graphene optical conductivity, $\sigma_{Gra}(\omega)$, is then given by the sum of the intraband and interband terms:[3] $\sigma_{Gra}(\omega) = \sigma_{intra}(\omega) + \sigma_{inter}(\omega)$. Here, we consider only the intraband absorption, which is the relevant term in the THz region[5,6] and we calculate the SLG conductivity according to the Drude model:[7]

$$\sigma_{Drude}(\omega) = \sigma_{DC}\frac{1}{1 - i\omega\tau}$$

Where τ is scattering time, $\sigma_{DC} = \frac{2e^2}{h}|k_F|v_F\tau$ is the static conductivity of Dirac fermions in SLG, with $v_F = 1 \times 10^6$ m/s the Fermi velocity, k_F the Fermi momentum, defined as $k_F = \sqrt{\pi n}$ and n, the 2d electron gas density of the Dirac system. τ is related to the mobility μ through the relation: $\tau = \frac{\mu E_F}{e v_F}$, where $E_F = \hbar v_F|k_F|$ is the Fermi Energy. The 3d conductivity for N-layers graphene (NLG) can be written as[3] $\sigma_{GRA} = \frac{N * \sigma_{Drude}(\omega)}{d}$, where d=0.335 nm is the SLG thickness, and N=7 for

our IG configuration. For the graphene ink reflector, we assume N=50 as discussed in Ref.[8] The effective complex dielectric function for the system comprising NLG on a dielectric substrate with refractive index n_{sub} is:

$$\varepsilon_{NLG}(\omega) = 1 + \frac{i\sigma_{NLG}(\omega)}{(n_{sub}+1)\varepsilon_0\omega}$$

The complex refractive index Ñ= $n_{NLG} + ik_{NLG}$ is extracted from:

$$n_{GRA} = Re(\sqrt{\varepsilon_{GRA}(\omega)}) \qquad \text{(eq. S1)}$$

$$k_{GRA} = Im(\sqrt{\varepsilon_{GRA}(\omega)}) \qquad \text{(eq. S2)}$$

In our 3d simulation model, NLG is treated as a transition boundary condition whose Ñ is defined from Eqs. S1,2, calculated using the parameters: n_{sub} =3.425, E_F=200 meV, μ=1000 cm^2/Vs, leading to τ=20 fs and σ_{DC}=0.43 mS, for SLG's contribution to the total conductivity. The nominal thickness, needed for the computation treatment of the interface, is set to 50 nm in EG and 2.4 nm for IG, accounting for the total effective thickness of 7 layers of 0.335 nm-thick SLG. The resulting n_{NLG}, k_{NLG} are shown in Fig. S1.

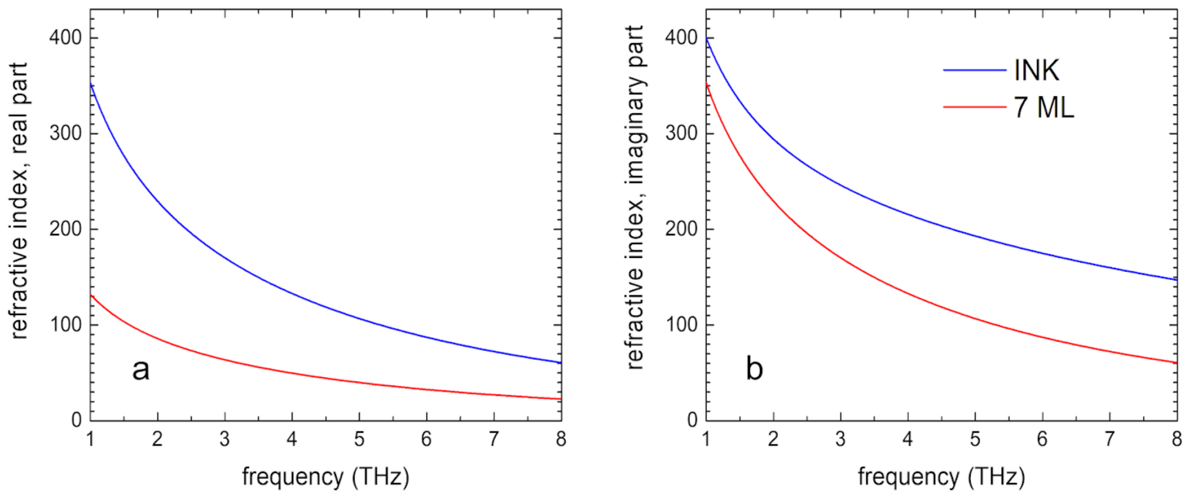

Figure S1: NLG complex refractive index. **(a)** *n*; **(b)** *k* from Eqs. S1,2 assuming E_F=200 meV, μ=1000 cm^2/Vs, for the 7LG (red) and for the printed graphene film (blue).

Section III. Intracavity Intensity in Graphene-Integrated Random Scatterers

To investigate the effect of 7LG on a single hole reflectivity, we perform simulations of a 200μm wide, 450μm long section of a RL cavity at 3.2THz. When there is no hole, the reflectance at the injection port is negligible (reflection coefficient, $r \sim 1.5\times10^{-6}$) and no standing wave pattern is observed (Fig. S2a). When a 5μm radius hole is introduced, a standing wave pattern is generated and the reflectance rises to $r \sim 6.71\times10^{-4}$ (Fig. S2b). When the hole is coated with a 7LG having n_G = 65 and k_G = 175, the standing wave pattern is reduced and reflectance decreases to r ~ 4.35×10^{-5},

i.e.~6% of the uncoated hole reflectance (Fig. S2c). The reduction of reflectance is always observed, aside for some extreme combinations of n_G and k_G (Fig. S2d). Hence, hole reflectance can be tuned by changing the optical properties of the MLG coating.

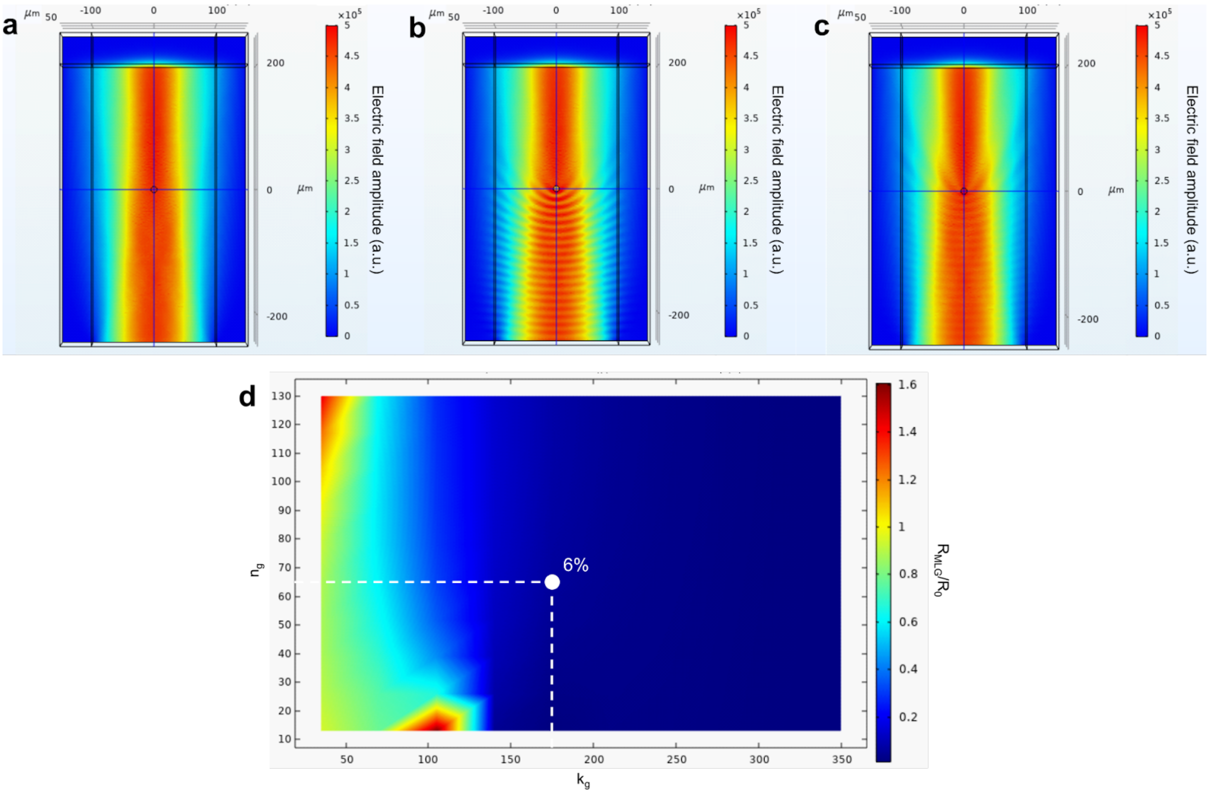

Fig. S2: a. Top view of simulated electric field amplitude in a 200µm wide, 450µm long section of a 10µm thick RL WG without holes, at 3.2THz. b. Top view of the simulated electric field amplitude in a 200µm wide, 450µm long section of a 10µm thick RL WG when a single 10µm radius hole is opened in the top Au top contact, at 3.2THz. c. Top view of the simulated electric field amplitude in a 200µm wide, 450µm long section of a 10µm thick RL WG when a single 10µm radius hole, coated with our 7LG, is opened in the top Au top contact, at 3.2THz. d. Reflectivity map, normalized to the reflectivity of the hole without coating, of a single hole coated with a 7LG with n_G and k_G at 3.2THz. For the 7LG used in our structure, a ~94% reduction in single hole reflectivity is observed.

IV. External graphene RLs

To corroborate our analysis, we test the EG configuration on 10 random QCLs with different sizes and surface pattern design, from three fabrication runs. The sensitivity of the spectral emission and intermode beating is more pronounced in multimodal lasers.

We now compare simulations and experiments on the two most representative samples, analyzed with the same approaches outlined in the main text.

a- Random QCL sample EG1

The first 2d resonator (sample EG1) comprises 80 holes with r=5 µm diameter, randomly distributed on a L=325 µm size area, corresponding to $\frac{r}{a} = 13.7\%$, where $a = \frac{L}{\sqrt{N}}$is the average inter-holes distance, enclosed in external irregular borders to prevent geometrical whispering-gallery-like or Fabry-Perot-like modes with high quality factors (Q). Fig. S3a compares the numerically calculated Q for the two configurations: 1) a random QCL with no graphene in the holes (pristine); 2) random QCL coupled with EG 50 µm from the top contact. The Q distribution is very similar in the two cases, aside from 4 eigenmodes ~3.116 THz, where Q is almost twice the pristine values. A closer look at the E-field distribution of the specific eigenmode in Fig. S2b indicates that the electric field is enhanced at the border closer to the holes where multiple scattering takes place (dotted rectangular area), hinting that the irregular frame of the resonator might contribute to and explain the Q increase.

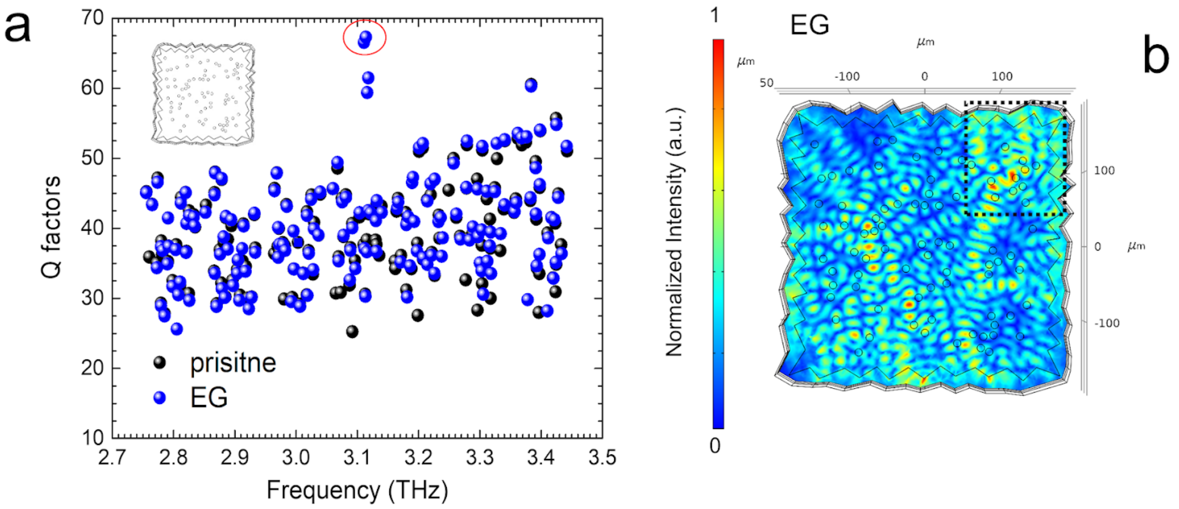

Figure S3: Numerical simulations of sample EG1. (a) Simulated Q for a random resonator having a total area of 0.106 mm^2 (area filling fraction 6%) and a 2d arrangement of 80 holes randomly distributed on a square area with $L = 325\ \mu m$ in the pristine (black) and EG (blue) configuration. Inset: resonator schematics. **(b)** Electric field distribution of ~3.117 THz eigenmode highlighted with the red circle in (a) for EG, calculated at the half height of the mesa (5 µm below the top surface). The dotted rectangular area outlines the resonator region where the multiple scattering, responsible for the higher Q of this specific mode, takes place.

Figure S4 plots L-J-V (S4a), spectral emission (S4b-c) and intermode beatnote (BN) maps (S4d-e), measured for sample EG1 in the two configurations. The spectral lines emitted at the same bias points are different, with the appearance of two additional random modes at 3.089 and 3.117 THz in EG (green area in Figs. S3b-c). The BN maps, acquired in the pristine (Fig. S4d) and EG (Fig. S4e) configurations, reveal single BNs in both cases, with the BN in the EG configuration (~8GHz) at a

frequency lower than in the pristine sample (~19.5 GHz), and similar intensity and persistence. The presence of the single BNs is ascribed to the beating of modes, showing different spacings as an effect of the graphene-induced modified intracavity field.

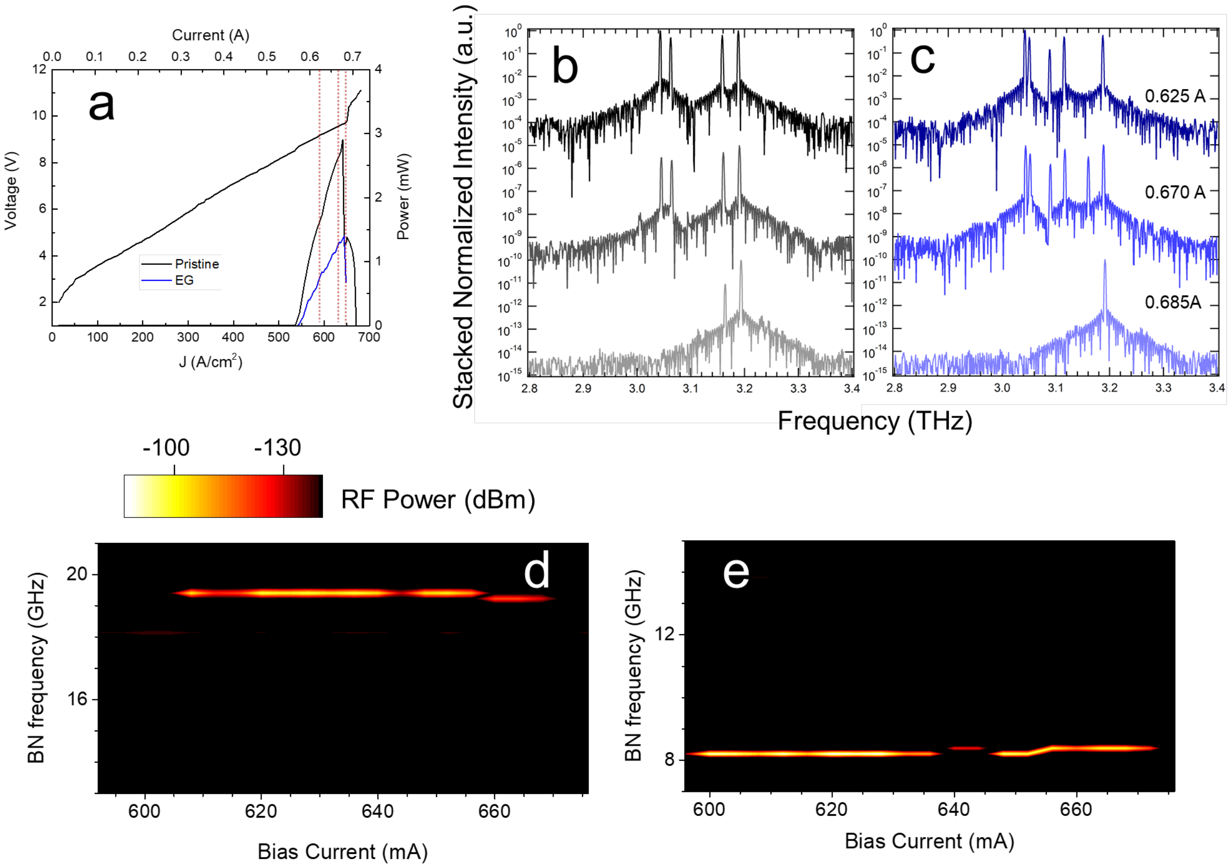

Figure S4: Experimental analysis of sample EG1. (a) J–V and J–L characteristics of EG1 RL, measured at a heat-sink temperature of 18 K while driving the lasers in quasi-CW mode with a pulse width of 100 µs and a repetition rate of 10 kHz (50%-duty cycle) in a vacuum environment, for pristine (black) and EG (blue) configurations. **(b-c)** FTIR stacked spectral emission of the EG1 RL in pristine (b) and EG (c) configurations, at the 3 driving currents indicated as red vertical dotted lines in (a): from top to bottom, 625mA (dark blue), 670 mA (blue), 685 mA (light blue). **(d-e)** Intermode beatnote (BN) maps measured in the (d) pristine device and (e) EG configuration. The BN signal is extracted from the bias line with a bias-tee and recorded with a radio frequency (RF) spectrum analyzer (RBW: 5 kHz, video bandwidth (VBW): 5 kHz, sweep time (SWT): 20 ms, RMS acquisition mode). All measurements in (b-e) are performed in CW, at a fixed heat sink temperature of 18 K.

The two BNs have comparable linewidths (LWs) (Fig. S5a) ranging between 50 and 300 kHz. The total radiative losses, calculated for the random design in Fig. S4b, show an overall decrease over the entire 2.8-3.4 THz spectral range for EG in comparison with the pristine. A very low loss range is expected ~3.1 THz for EG, where the emission spectrum shows the spectral lines not emitted in the pristine case.

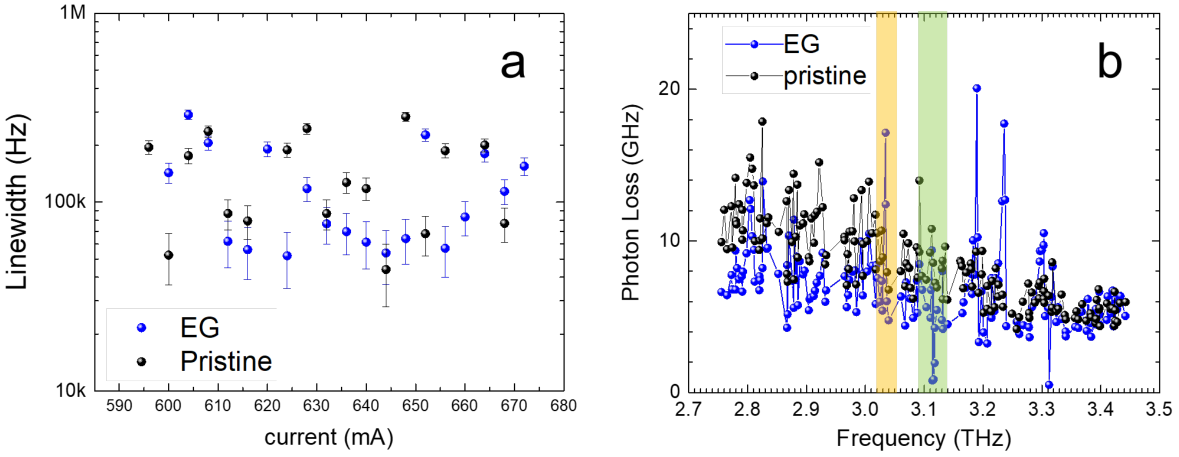

Figure S5: Mode locking of sample EG1. (a) Intermode BN linewidths as a function of driving current for the ~19.5 and ~8 GHz BNs measured in the pristine (black) and EG (blue) configurations, for the EG1 RL. (b) Photon loss rate calculated for pristine (black) and EG (blue) configurations. The yellow region indicates the frequency range responsible for the single and narrow BNs in the map. The green region indicates the region where the EG configuration results in lasing modes not visible in the pristine case.

b- Random QCL sample EG2

The second 2d resonator (sample EG2, Fig. S6a) comprises 12 holes with r=5 μm, distributed on a L=225 μm size area, corresponding to $\frac{r}{a} = 7\%$, A numerical study on Q_{tot} (Fig. S6b) reveals a distinctively different behavior with respect to sample EG1. Q_{tot} values in both pristine and EG configurations are almost one order of magnitude larger than the corresponding cases in EG1. A closer look to the electric field distribution (Fig. S6c) of the computed eigenmodes reveals that the mesa border and the Cr coated frame deeply affect the optical modes confinement. The oscillating Q-factors in the EG case (Fig. S6b) are likely an artifact of the calculation since the corresponding E-field 2D distributions remain the same for different frequencies, aside from changes in the E-field strength. (Fig. S6b). The effective Q_{tot} distribution could be obtained by averaging the set of Q_{tot} around each specific frequency solution set point (i.e. 2.8, 2.9,…,3.4 THz).

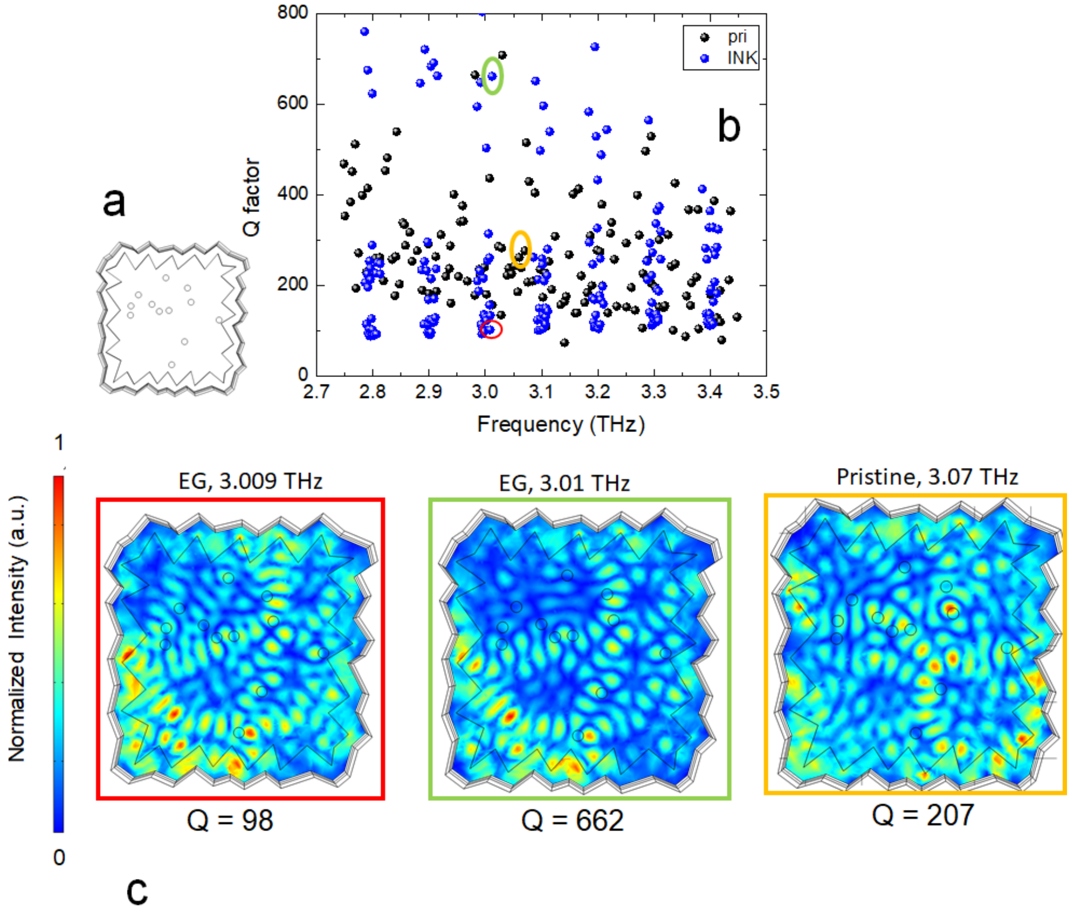

Figure S6: Numerical Simulations of sample EG2. (a) Random resonator design of sample EG1, with total area ~0.0506 mm^2 (filling fraction 1.7%), a 2d arrangement of 12 holes randomly distributed on a square area with $L = 225$ μm. **(b)** Simulated Q for pristine (black) and EG (blue) configurations for sample EG2. (c) Electric field distribution at the eigenmodes: ~3.009 THz (right), ~3.01 THz (centre) in the EG configuration, and at ~3.07 THz (left) in the pristine configuration, highlighted respectively with red, green and orange circles in (b).

Figure S7 plots the L-J-V (S7a), the spectral emission (S7b-c) and the intermode BN maps (S7d-e) for sample EG2 in the two configurations. The spectra acquired at the same bias points show visible differences, with the EG configuration featuring a much denser multimodal emission. Here, the BN maps show the most remarkable differences. In the EG case, three distinct single BNs are visible, at ~12, ~21, ~25 GHz (Fig. S7e), absent in the pristine architecture (Fig. S7d). The spectral lines, whose spacing matches the BN frequency, are present only in the emission spectra collected in the EG configuration (Fig. S6c), meaning that the graphene here promotes mode correlation.

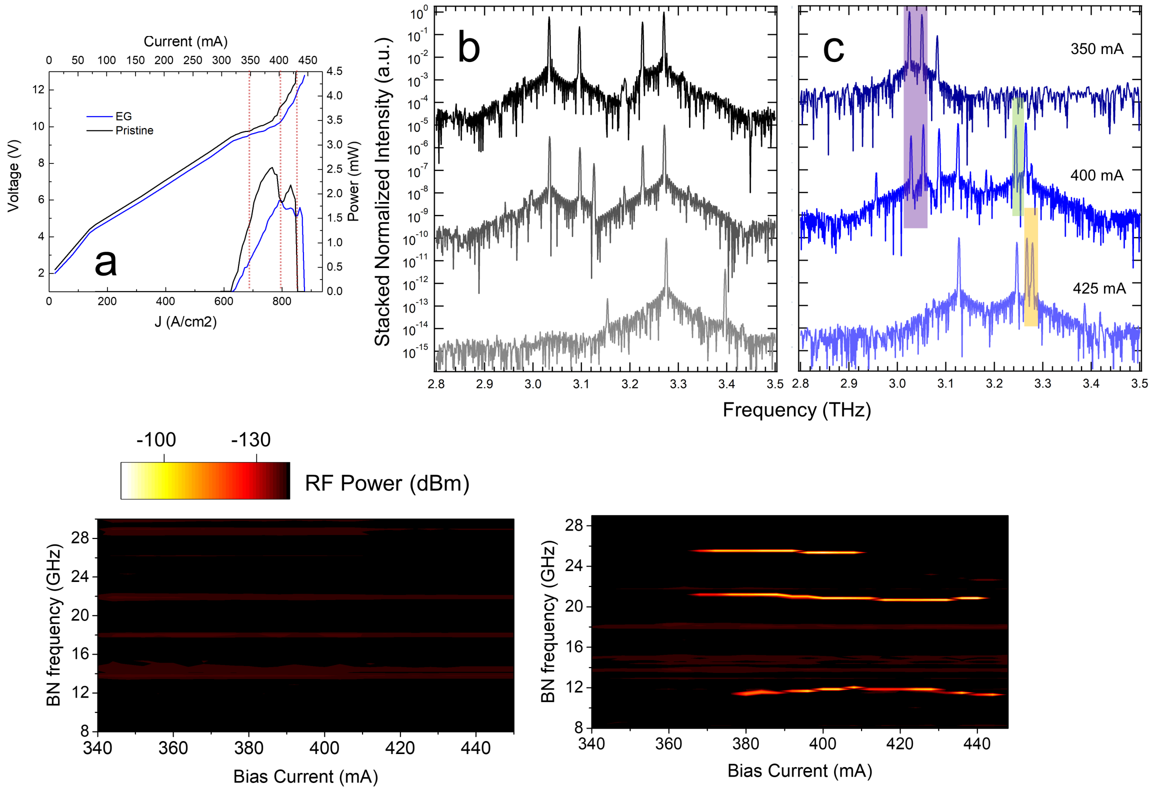

Figure S7: Experimental analysis of sample EG2. (a) J–V and J–L characteristics of EG2 RL, measured at a heat-sink temperature of 18 K while driving the lasers in quasi-CW mode with a pulse width of 100 µs and a repetition rate of 10 kHz (50%-duty cycle) in a vacuum environment, for pristine (black) and EG (blue) configurations. The dotted vertical lines indicate the driving currents at which the spectra of (b-c) are measured. **(b-c)** FTIR stacked spectral emission of EG2 RL in (b) pristine and (c) EG configurations, at the 3 driving currents indicated as red vertical dotted lines in (a): from top to bottom, 350mA (dark blue), 400 mA (blue), 425 mA (light blue). **(d-e)** Intermode BN maps measured in (d) pristine and (e) EG configurations. The BN signal is extracted from the bias line with a bias-tee and recorded with an RF spectrum analyzer (RBW: 5 kHz, video bandwidth (VBW): 5 kHz, sweep time (SWT): 20 ms, RMS acquisition mode). All measurements in (b-e) are performed in CW, at a fixed heat sink temperature of 18 K. The colored areas in (c) highlight the spectral lines whose spacing match the single BN in (e), located at ~12 GHz (yellow), ~21 GHz (green), and ~25 GHz (purple).

The linewidth analysis (Figs. S8a-c) reveals a visible broadening of the intermode BN linewidths of the ~12 and ~21 GHz BNs. In the first case, the linewidth ranges from 700 kHz to 10 MHz and then decreases again at 600 kHz. In the second case, it increases from 300 kHz to 20 MHz, then decreasing again at 250 kHz. This latter effect is not seen for the broader ~25GHz BN, which is first 750 kHz wide, then reduces at 150 kHz and increases again at 500 kHz, suggesting a correlation between the mode interaction from which the beating originates. The lasing lines responsible for the these two BNs are closer in frequency, hinting that the observed mode locking is favored when multiple lasing peaks cluster around each other, as typical for RLs.[7]

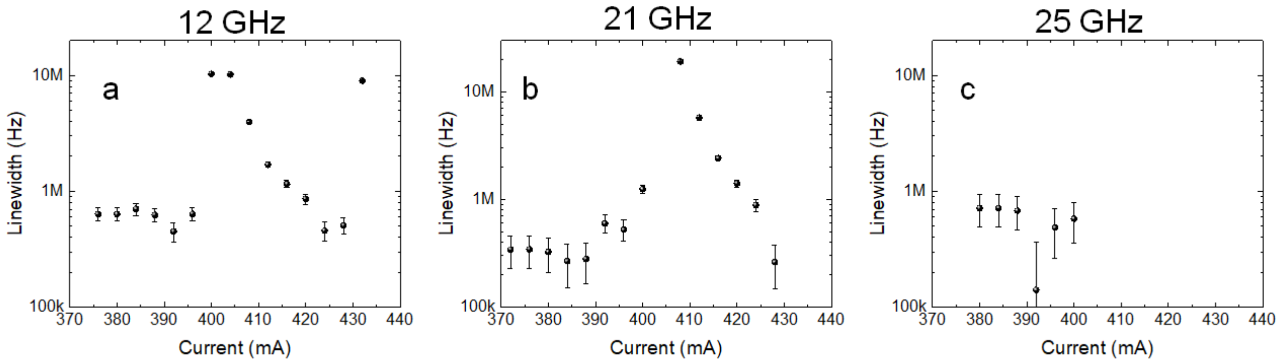

Figure S8: Linewidth analysis of EG configuration. Intermode BN linewidths as a function of driving current for (a) ~12, (b) ~21, (c) ~25 GHz BNs, visible only with the EG mirror for the EG2 RL.

V. Integrated graphene RLs

We design, fabricate and test optically a set of different QCLs in the IG configuration, implementing on the same chip a set of two identical QCLs: IG device and pristine counterpart. These IG samples have the same size (L=325 mm, total area of 0.105 mm^2), and $\frac{r}{a}$ ranging from ~ 9 to 26%.

a- Random QCL sample IG1, r/a= 8.95%

The first 2d resonator (sample IG1, Fig. S9a-b) comprises 94 holes with r=3µm, corresponding to $\frac{r}{a}$ = 8.95%. A numerical study of Q_{tot} (Fig. S9c) and photon loss rate (Fig. S9d) shows that the integration of graphene is expected to have a moderate influence on the laser behavior. The lower Q-distribution spread ($<Q_{tot,IG}>$ = 34.4 ±4.4 for IG; $<Q_{tot,pri}>$= 36.3 ±5.4 for pristine configuration) and the increased radiative losses in the IG case resemble and confirm the behavior described in the main text.

The experimental analysis of sample IG1 is in Fig. S10, showing L-J-V (Fig. S10a), spectral emission (Figs. S10b-c) at the bias currents indicated by the vertical dotted lines, and the intermode BN maps (Figs. S10d-e), measured for (d) IG and (e) pristine cases.

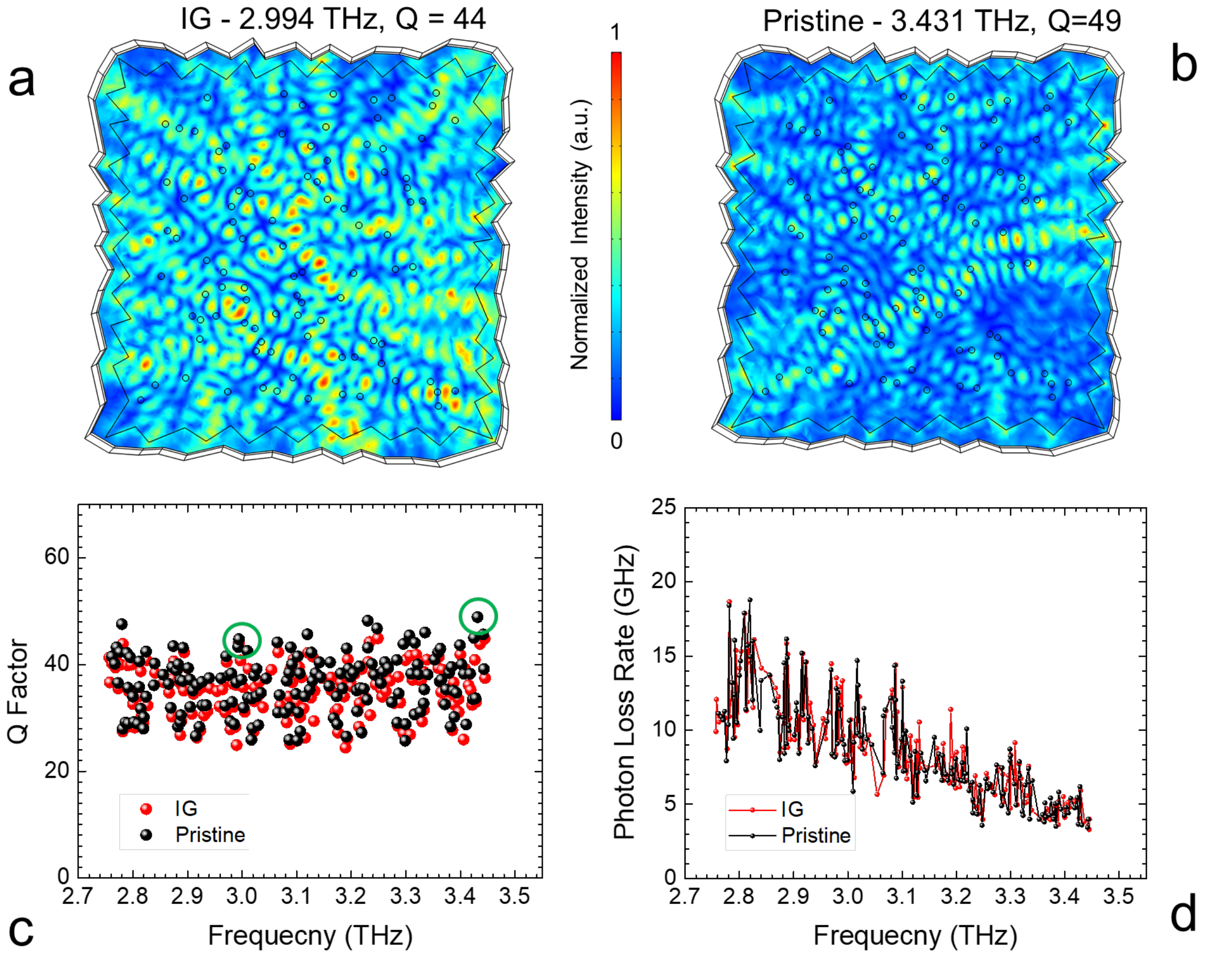

Figure S9: Numerical simulations of sample IG1. (a-b) Electric field distribution of IG1 resonator at the eigenmodes: (a) 2.994 THz for IG, and (b) 3.431 THz for the pristine device. **(c)** Q and (d) photon loss rate numerically calculated for pristine (black) and IG (red) resonator configurations for sample IG1 in (a-b). The green circle in (c) indicates the specific eigenmodes corresponding to the E-field maps of (a-b), chosen as the highest Q for the two cases.

The spectra, acquired at the same driving currents, have differences, with the IG configuration featuring a larger number of lasing modes along the entire operational range, particularly evident at the higher current (710 mA), where the IG laser emits ~17 modes against the 5 collected in the pristine resonator. The BN maps show a single and narrow (up to ~110kHz) BN at ~11.3GHz in the IG case, persisting over more than 60 mA, while an extremely broad BN (> 100 MHz) is retrieved in an identical pristine random resonator. At larger currents a set of multiple, narrow (130 kHz) BNs are retrieved, signature of the locking of the multiple random modes arising from graphene integration. The analysis of the linewidth of the BN at ~11.3GHz (Fig. S10f), as a function of bias current, reveals linewidth values ranging from 116 kHz (Fig. S10g) to 316 kHz (Fig. S10h), signature of phase-locking of RL modes from which it arise.

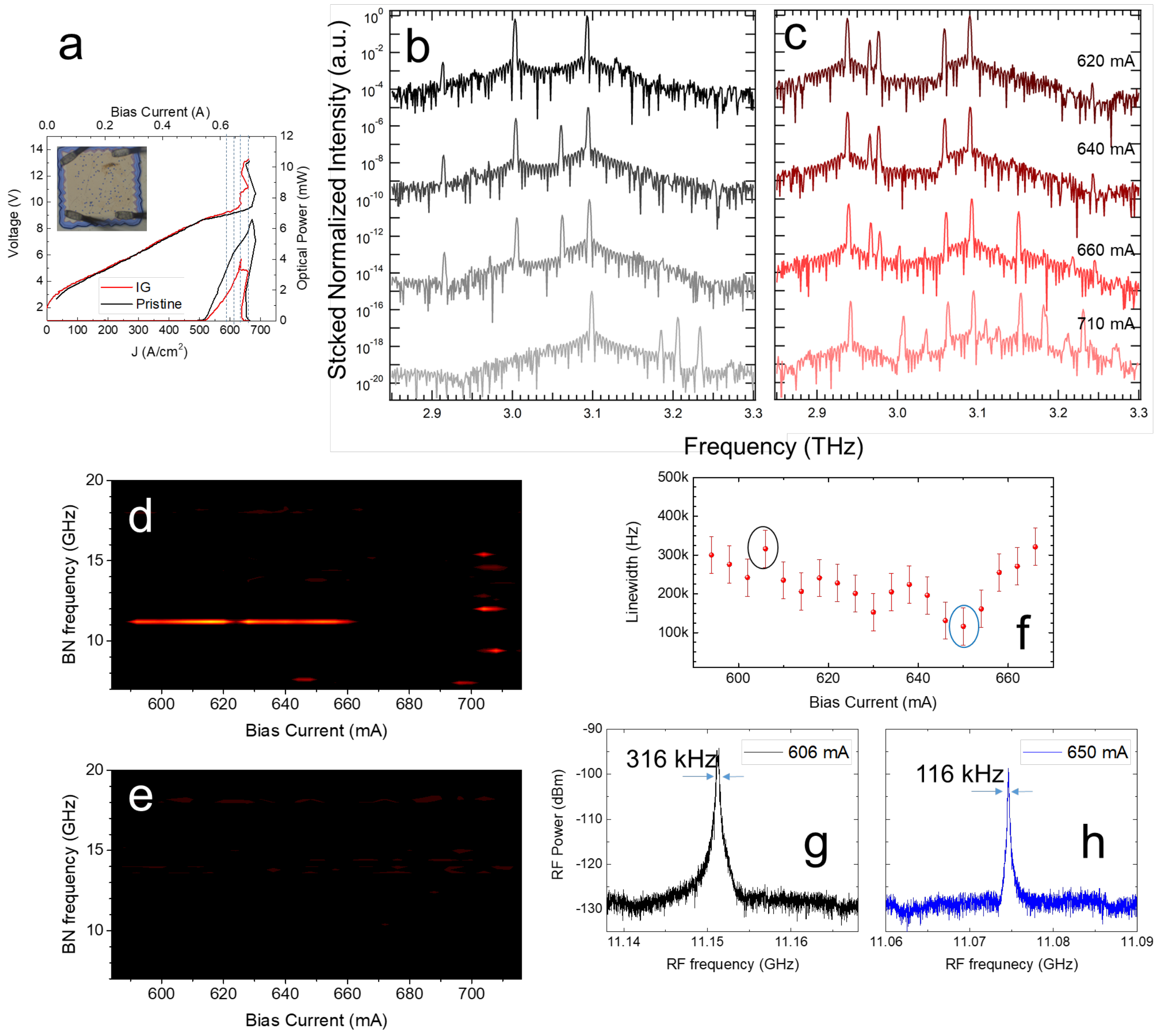

Figure S10: Experimental analysis of sample IG1. (a) J–V and J–L characteristics of IG1 RL, featuring two random QCLs of Fig. S9 (pristine and IG configuration on the same chip sample), measured at a heat-sink temperature of 18 K while driving the lasers in quasi-CW mode with a pulse width of 100 µs and a repetition rate of 10 kHz (50%-duty cycle) in a vacuum environment, for pristine (black) and IG (red) configurations. The inset shows the optical microscope image of the IG laser **(b-c)** FTIR stacked spectral emission of (b) pristine and (c) and IG RLs, at the 4 driving currents indicated as light blue dotted lines in (a): from top to bottom, 620, 640, 660, 710 mA. **(d-e)** Intermode BN maps in the (d) IG and (e) pristine configurations. The BN signal is extracted from the bias line with a bias-tee and recorded with an RF spectrum analyzer (RBW: 5 kHz, VBW: 5 kHz, SWT: 20 ms, RMS acquisition mode). The colored light blue area in (d) highlights the spectral lines whose spacing matches the single BN in the IG case in (d), at ~11.3 GHz. **(f)** Intermode BN linewidths as a function of driving current for the ~11.3 GHz BN, visible in (d) only for the IG case. **(g-h)** highest and lowest linewidth intermode BN traces at (g) 606 and (h) 650 mA, corresponding to the (g) black and (h) blue circles in (f). All measurements in (b-h) are performed in CW, at fixed heat sink temperature of18 K.

b- Random QCL sample IG2, r/a= 22%

The second 2d resonator (sample IG2, Fig S11a-b) comprises 576 holes with r=3µm, corresponding to $\frac{r}{a} = 22\%$. Numerical simulations (Figs. S11a-d) reveal the overall decrease of Q_{tot} in IG resonators with different holes arrangement, with the simultaneous narrowing of the Q_{tot} distribution (Fig. S11c). Q_{tot} are distributed around $<Q_{tot,pri}> = 36 \pm 5$ in the pristine configuration, and $<Q_{tot,IG}> = 29 \pm 3$ in the IG case, calculated over the 2.75-3.45 THz spectral range. The photon

loss rate (Fig. S11d) is also affected by graphene integration, without a distinct increasing/decreasing behavior amongst the two configurations.

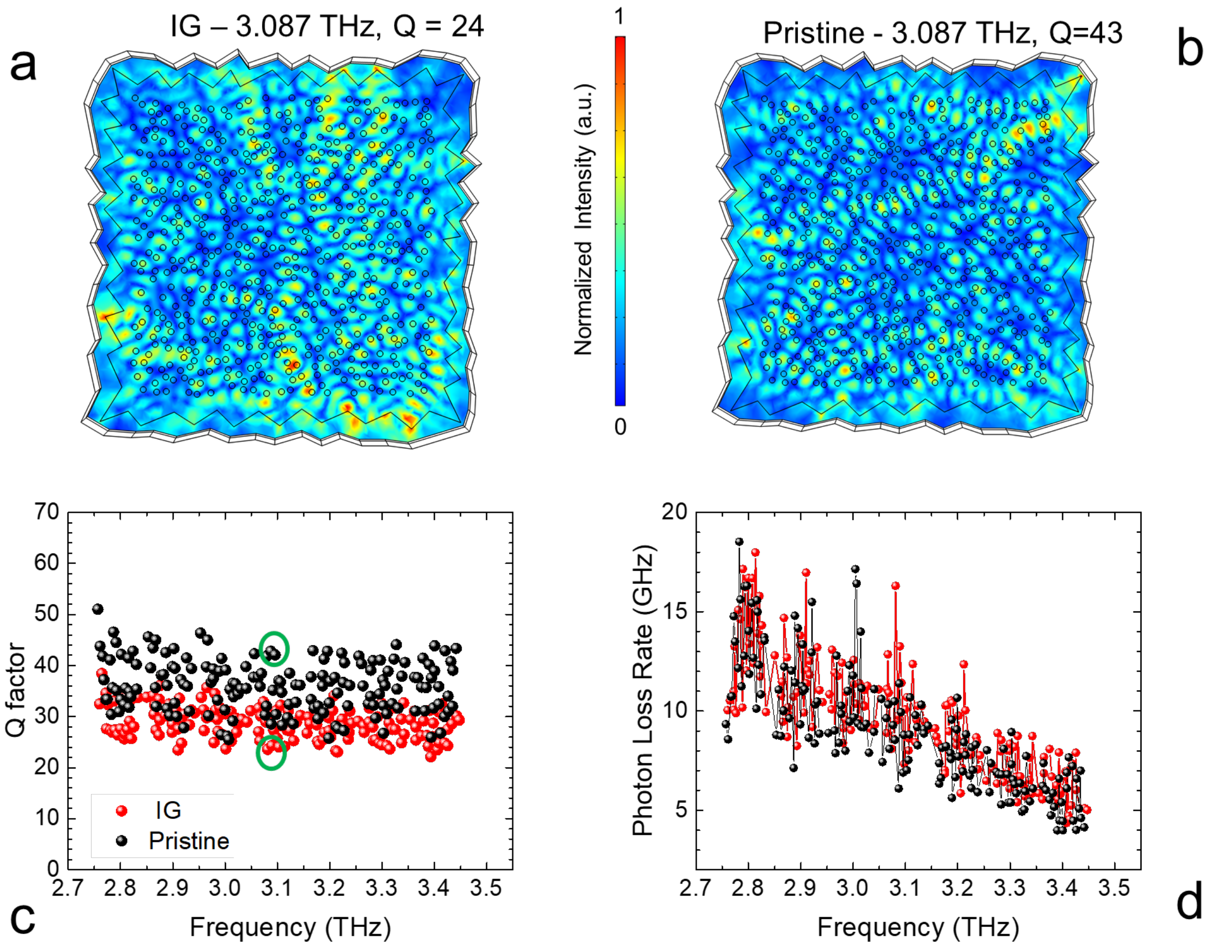

Figure S11: Numerical simulations of sample IG2. (a-b) Electric field distribution of IG2 RLs, calculated at the 3.087 THz eigenmodes for (a) IG and (b) pristine configuration. **(c)** Q and **(d)** photon loss rate numerically calculated for pristine (black) and IG (red) configurations of (a-b). The green circles in (c) indicate the specific eigenmodes corresponding to the electric-field maps of (a-b).

The experimental results for sample IG2 are in Fig. S12, reporting L-J-V (Fig. S12a), spectra (Figs. S12b-c) acquired at 4 bias points chosen in correspondence of the relevant regions of the BN map (Figs. S12d-e): before (590 mA), within (620, 655 mA), and after (690 mA) the intervals in which the IG configuration induces a single and narrow BN. Similarly to the previous IG lasers, the IG device has a stronger multimodal emission and richer intermodal BN maps. The IG map features 3 single narrow BNs (100-150 kHz linewidth, Fig. S12f), at ~20, 14, 6GHz, respectively. Figure S12f shows the linewidth analysis for the ~20 and ~14 GHz BNs, arising respectively from the laser modes located at 3.151 and 3.131 THz. The BN ~6 GHz originates from RF down-conversion of the other two BNs, hence it is not related to the direct beating of two lasing modes. Thus, its linewidth (not shown) is not relevant. When graphene is not integrated in the holes, such a random

resonator does not show any mode correlation effect and an intermode BN is not retrieved (Fig. S12d). The intermodal spectrum is dominated by a > 100 MHz broad band.

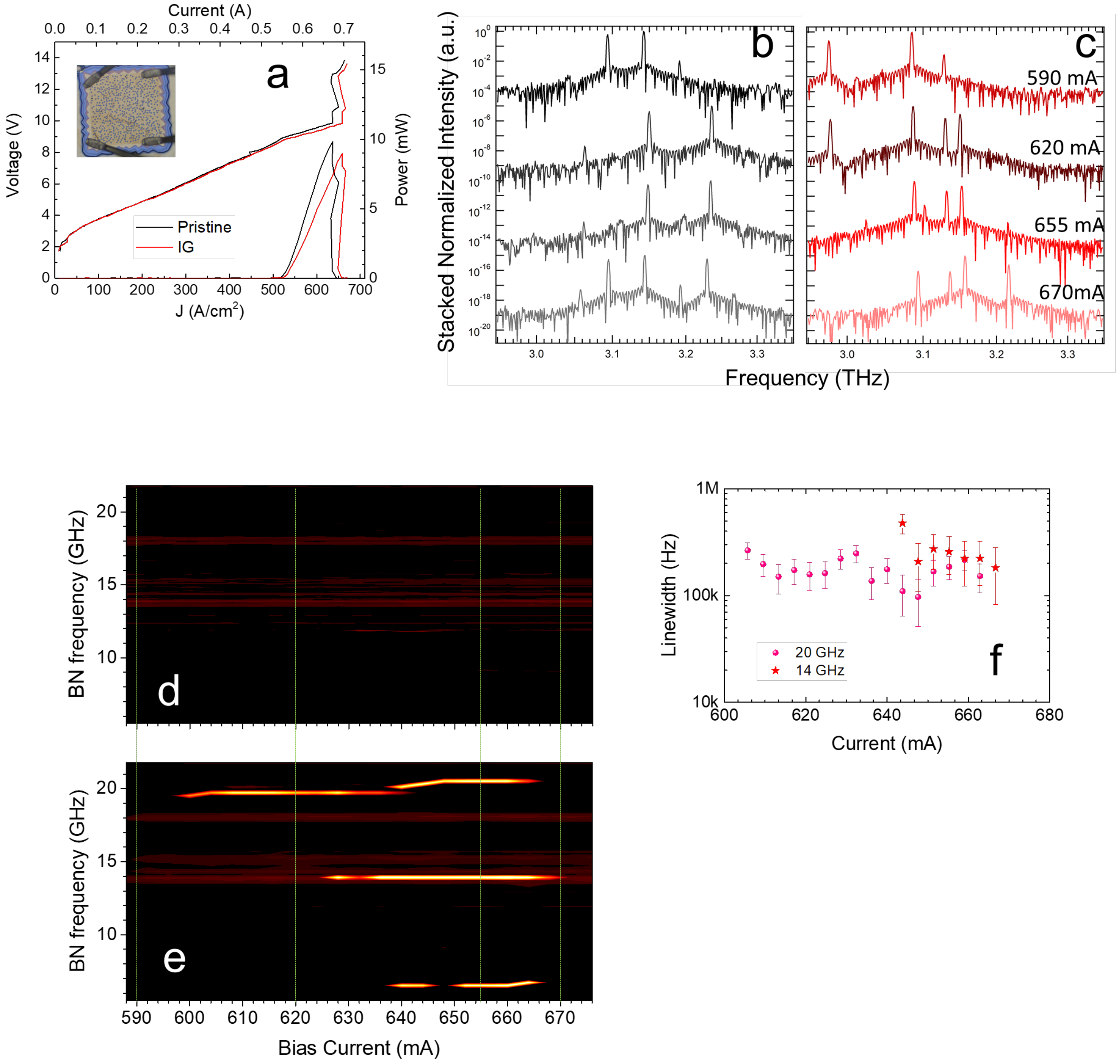

Figure S12: Experimental analysis of sample IG2. (a) J–V and J–L characteristics of IG2 RL, featuring the two random QCLs of Fig. S10a-b (pristine and IG configuration on the same chip sample), measured at a heat-sink temperature of 18 K while driving the lasers in quasi-CW mode with a pulse width of 100 μs and a repetition rate of 10 kHz (50%-duty cycle) in a vacuum environment, for pristine (black) and IG (red) configurations. The inset shows the optical microscope image of the IG RL. **(b-c)** FTIR stacked spectral emission of (b) pristine and (c) IG RLs, at 4 driving currents: from top to bottom, 590, 620, 655, 670mA. **(d-e)** Intermode BN maps measured in the (d) pristine device and in (e) the IG configuration. The BN signal is extracted from the bias line with a bias-tee and recorded with an RF spectrum analyzer (RBW: 5 kHz, VBW: 5 kHz, SWT: 20 ms, RMS acquisition mode). The vertical green lines indicate the bias set points of the spectra in (b-c). The light green and yellow areas in (c) highlight the spectral lines whose spacing matches the single BNs visible in the IG case in (e), at ~20 (green) and ~14 GHz (yellow). All measurements in (b-e) are performed in CW, at a fixed heat sink temperature of 18 K. **(f)** Intermode BN linewidths as a function of driving current for the ~20 (pink circles) and ~14 GHz (red stars), visible only for the IG case in the RF map in (d).

c- Random QCL sample IG3, r/a= 18.6%

The third 2d resonator (sample IG3, Fig S13a-b) comprises 146 holes with r=5μm, corresponding to $\frac{r}{a}$ = 18.6%. The simulated Q_{tot} (Fig. S13c) and photon loss (Fig. S13d) confirm the same overall decreasing and narrowing of the Q_{tot} - values distribution of IG resonators observed in the previous cases. Q_{tot} values are distributed around $< Q_{tot,PRI} >$ = 42 ± 8 in the pristine case, and $< Q_{tot,\mathrm{IG}} >$ = 33 ± 4 in IG. For this design, graphene integration has a much higher impact on the resonator modes in the upper part of the laser optical BW (>3.1 THz).

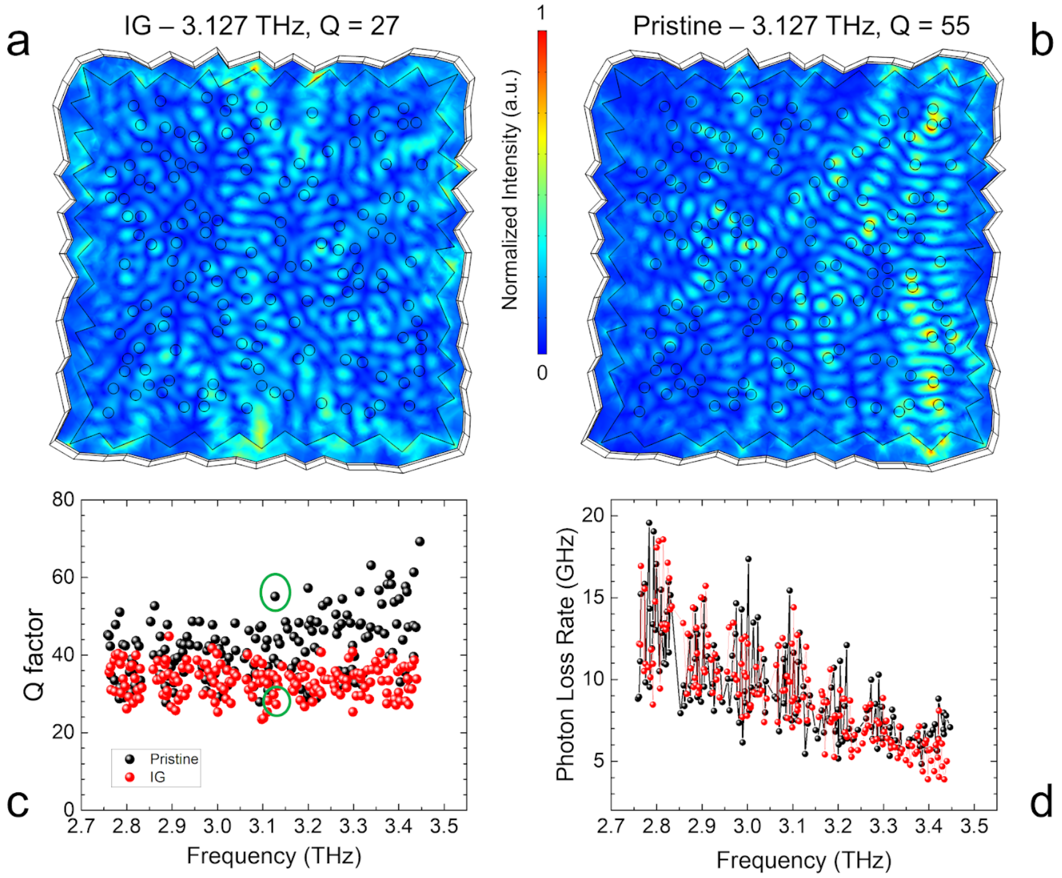

Figure S13: Numerical simulations of sample IG3. (a-b) Electric field distribution of IG3 RLs at the 3.127 THz eigenmodes for (a) IG and (b) pristine configurations. **(c)** Q and **(d)** and photon loss rate calculated for pristine resonator (black) and IG (red) configurations for sample IG3 in (a-b). The green circles in (c) indicate the specific eigenmodes corresponding to the E-field maps of (a-b).

For these modes the differences between EM field distributions (Figs. S12a-b) in pristine and IG configuration, and extrapolated Q (Fig. S13c) is more pronounced. The photon loss rate (Fig. S13d) is also affected by the graphene integration, without distinct increasing/decreasing behavior amongst the two configurations.

The experimental results of sample IG3 are in Fig. S14, showing L-J-Vs (Fig. S14a), spectral emission (Fig. S14b-c) at three driving currents: 600, 650, 700 mA. The spectral emission

in IG and pristine configurations is significantly different in terms of mode number and frequency. In IG, a denser multimodal spectrum is retrieved over the entire dynamic range up to the peak emission, where also the pristine device is significantly enriched. Both devices show the typical random mode proliferation at high biases, close to the peak power.

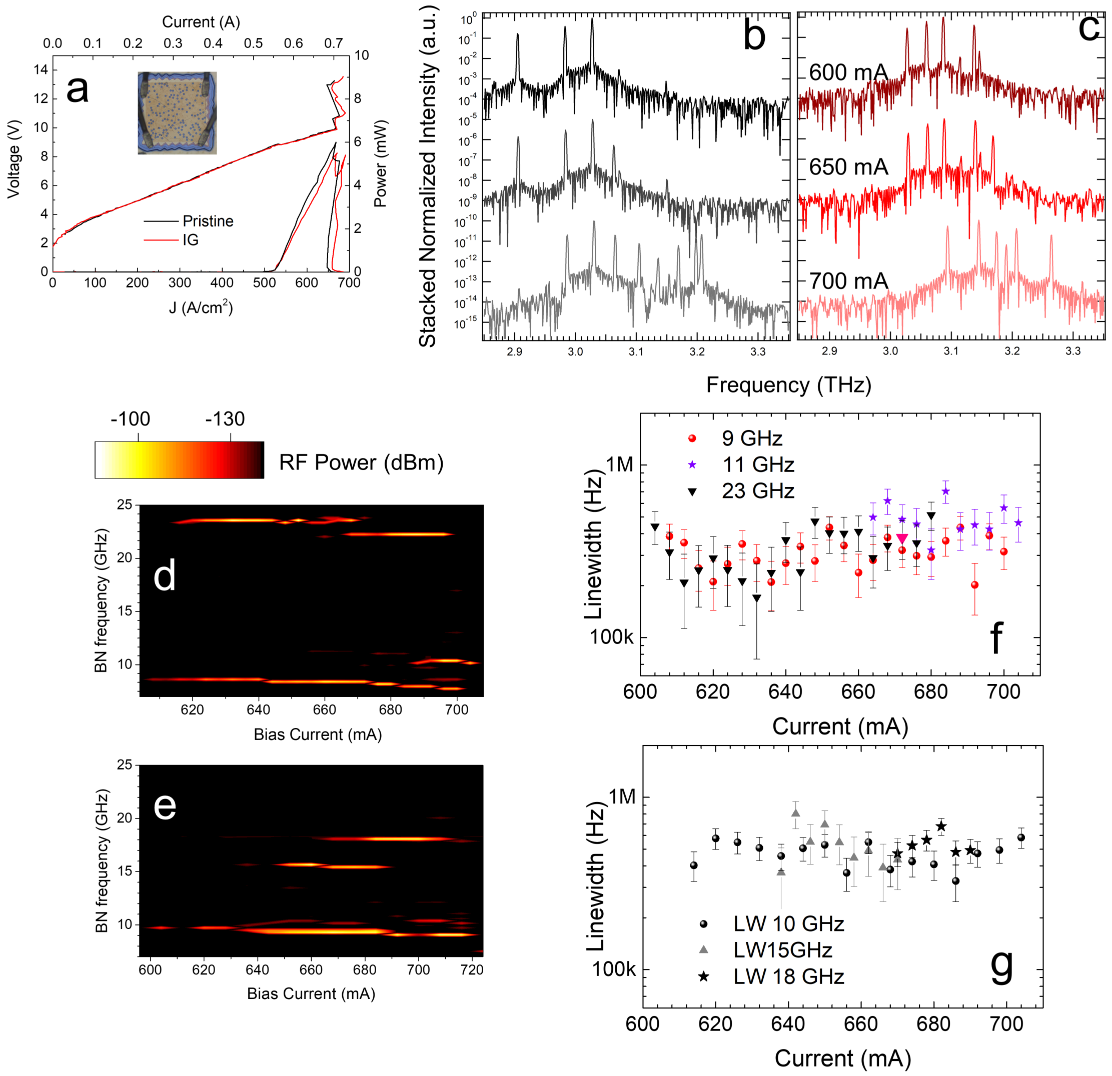

Figure S14: Experimental analysis of sample IG3. (a) J–V and J–L characteristics of IG3 RL, featuring the two random QCLs of Fig. S13 (pristine and IG configurations on the same chip sample), measured at a heat-sink temperature of 18 K while driving the lasers in quasi-CW mode with a pulse width of 100 µs and a repetition rate of 10 kHz (50%-duty cycle) in a vacuum environment, for pristine (black) and IG (red) configurations. The inset shows the optical microscope image of the IG RL **(b-c)** FTIR stacked spectral emission of (b) pristine and (c) IG RLs, at 3 driving currents: from top to bottom, 600, 650, 700mA. **(d-e)** Intermode BN maps measured in the (d) IG and (e) pristine configurations. The BN signal is extracted from the bias line with a bias-tee and recorded with an RF spectrum analyzer (RBW: 5 kHz, VBW: 5 kHz, SWT: 20 ms, RMS acquisition mode). All measurements in (b-e) are performed in CW, at a fixed heat sink temperature of 18 K. **(f)** Intermode BN linewidths as a function of driving current for ~23 (black triangles), ~11 (purple stars), ~9 GHz (red circles), in IG. **(g)** Intermode BN linewidths as a function of the driving current for ~18 (dark grey stars), ~15 (grey triangles), ~10 GHz (black circles), for the pristine case.

The BN map measured in the pristine device (Fig. S14e) shows three single BNs at ~10, 15 and 18 GHz, that are associated to the beating between the spectral lines in Fig. S13b located at 3.199 and

3.219 THz for ~10 GHz, 3.153 THz; 3.168 THz for ~15 GHz; 3.153 and 3.135 THz for ~18 GHz. Figure S14d show similar measurements performed on the IG device. Figs. S13f-g plot the linewidths (LW) of the three BNs for IG (Fig. S12f) and pristine (Fig. S12g) configurations. While in the first case the LW is in the 160–400 kHz range, with the exception of the 11 GHz BN (LW = 500 kHz), in the pristine configuration a much broader LW (600-900 kHz) is measured for all 3 BN frequencies.

d- Random QCL sample IG4, r/a= 13.7%

The fourth 2d resonator (sample IG4, Fig. S15a) has the same random arrangement of EG1, comprising 80 holes with r=5 μm, distributed on a L=325 μm size area, corresponding to $\frac{r}{a} = 13.7\%$. The simulated Q_{tot} (Fig. S15b) and photon loss rate (Fig. S15c) confirm the same overall decrease and narrowing of the Q_{tot} distribution of IG resonators. Q_{tot} values are distributed around $< Q_{tot,pri} > = 39 \pm 7$ in the pristine configuration, and $< Q_{tot,IG} > = 33 \pm 4$ in IG. Graphene integration into the scatterers holes is not affecting the photon loss rate in a unique way, in agreement with the results found in our different random designs.

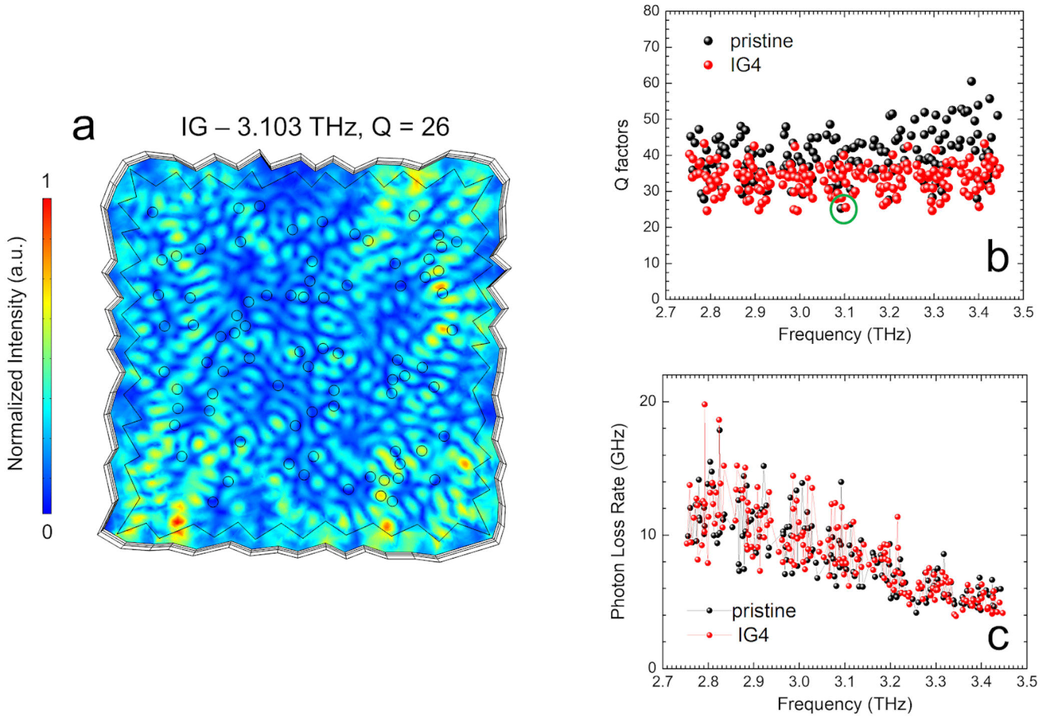

Figure S15: Numerical simulations of sample IG4. (a) Electric field distribution of IG4 RL at 3.103THz. **(b)** Simulated Q and **(c)** photon loss rate for pristine (black) and IG (red) configuration. The green circle in (b) indicates the specific eigenmode corresponding to the electric-field maps of (a).

The experimental analysis of sample EG4 is reported in Fig. S16, showing the L-J-Vs (Fig. S16a) and spectral emission (Figs. S16b-c) at three bias currents indicated inside the panel: 680, 700, 710

mA, corresponding to three driving current regions: before, within and above the measured BN (Figs. S16d-e).

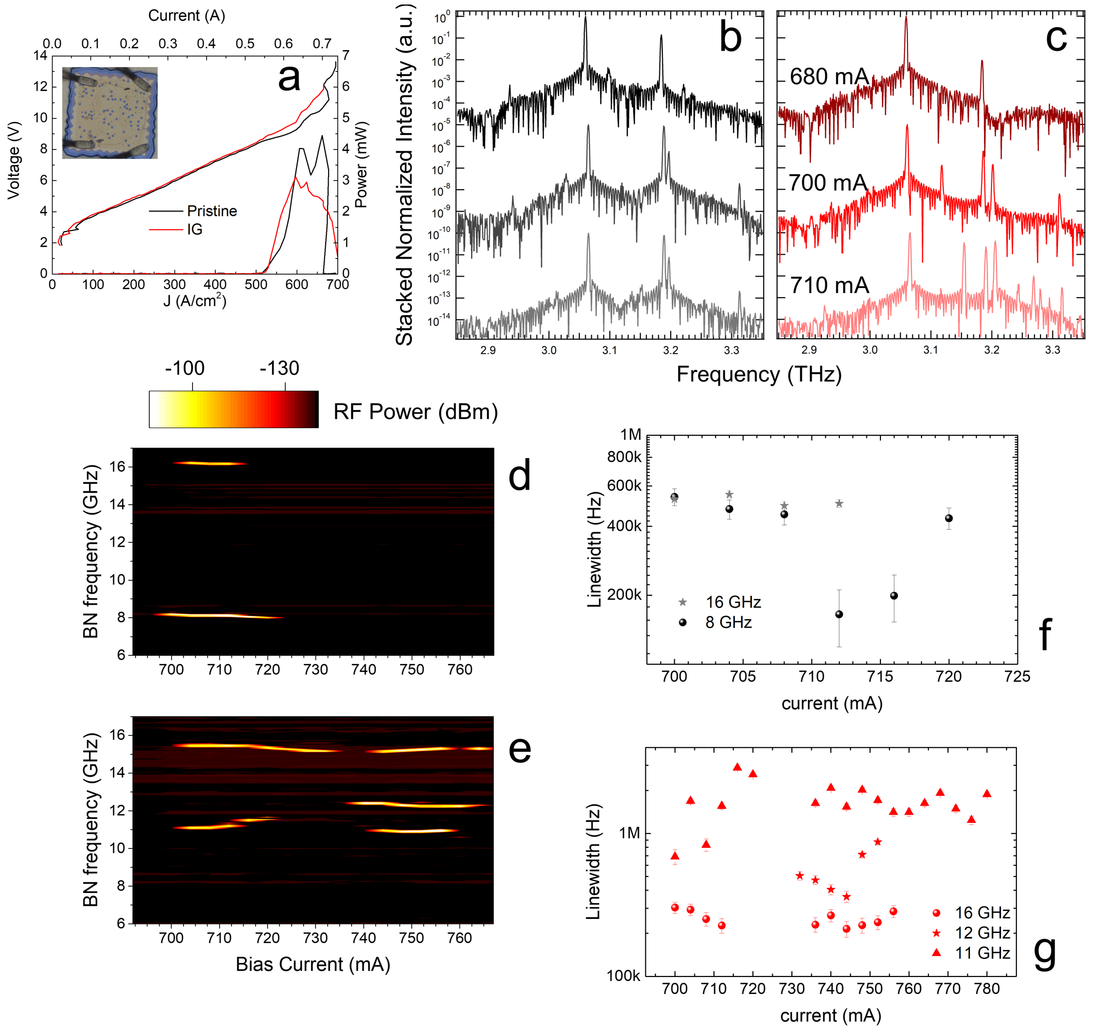

Figure S16: Experimental analysis of sample IG4. (a) J–V and J–L characteristics of IG4 RL, featuring the two random QCLs of Fig. S14 (pristine and IG configuration on the same chip sample), measured at a heat-sink temperature of 18 K while driving the lasers in quasi-CW mode with a pulse width of 100 µs and a repetition rate of 10 kHz (50%-duty cycle) in a vacuum environment, for pristine (black) and IG (red) configurations. The inset shows the optical microscope image of the IG RL. **(b-c)** FTIR stacked spectral emission of (b) pristine and (c) IG RLs, at 3 driving currents: from top to bottom, 680, 700, 710 mA. **(d-e)** Intermode BN maps measured in the (d) pristine and (e) IG device. The BN signal is extracted from the bias line with a bias-tee and recorded with an RF spectrum analyzer (RBW: 5 kHz, VBW: 5 kHz, SWT: 20 ms, RMS acquisition mode). All measurements in (b-e) are performed in CW, at a fixed heat sink temperature of 18 K. **(f)** Intermode BN LWs as a function of driving current for ~16 (red circles), 8 GHz (red stars) BNs for the pristine case. **(g)** Intermode BN LWs as a function of driving current for ~16 (dark grey stars), 12 (red triangles), 11 GHz (black circles) BNs for the IG case.

The spectral emissions of the two devices have some similarities at the lowest bias currents, then become significantly different at higher bias, where IG shows multimodal emission, absent in the pristine device. This behavior is well reflected by the appearance of several single BNs in the IG map at bias current well above 720 mA (Fig. S16d), not visible in the BN map (Fig. S16e) of the pristine device. Figures S16d-e reveal a variety of single BNs in both cases. The IG map (Fig. S16e)

shows three single BNs at ~11, 12, 16 GHz. The first two BNs are likely originating from the same spectral mode pair at 3.269 and 3.280 THz, visible in the spectrum acquired at 710 mA. The ~16 GHz BN can be ascribed to the beating of the intense modes at 3.190 and 3.205 THz in the spectra collected at 700 and 710 mA. In the pristine configuration two single BNs ~8 and ~16 GHz appear. The signal at ~8 GHz is associated to the beating between the spectral lines at 3.197 and 3.188 THz. This beating also originates at a high order harmonic ~16 GHz, as the precise replica at the second order of its frequency, and the persistence over approximately the same bias range, allow to confirm. We therefore compare the LW (Figs. 15f-g) of the ~8 GHz BN in the pristine configuration with that of the ~16 GHz BN in IG, as they arise from the same pair of beating modes. In the latter case, a LW ~200-300 kHz is measured, a factor of two narrower than that observed in the pristine configuration (~500-600 kHz).

e: EG Random Laser Performances

We evaluate the effect of graphene integration on the optical performance of a set of QCLs (Fig.S17) by estimating the main figures of merit as a function of the filling factor *r/a,* where *r* is the radius of the patterned surface holes and *a* the average intersite distance. The threshold density current J_{th} (Fig. S17a) is almost independent on *r/a* in both the pristine and IG cases, and it is only slightly higher than J_{th} of the Fabry-Perot QCL (410 A/cm^2) having the same active region design.[30] The comparison between slope efficiency (Fig. S17b), power extraction (Fig. S17c) and maximum wall plug efficiency (Fig. S17d) reveals, in all cases, an increase at *r/a* = 22%, followed by a decrease for *r/*a > 25%, as an effect of the enhanced optical confinement in the random system.[24] Our findings suggest an optimal *r/a* ~ 22%, as the best compromise between higher scattering strength in highly disordered media and loss increase in high hole density random photonic structures.

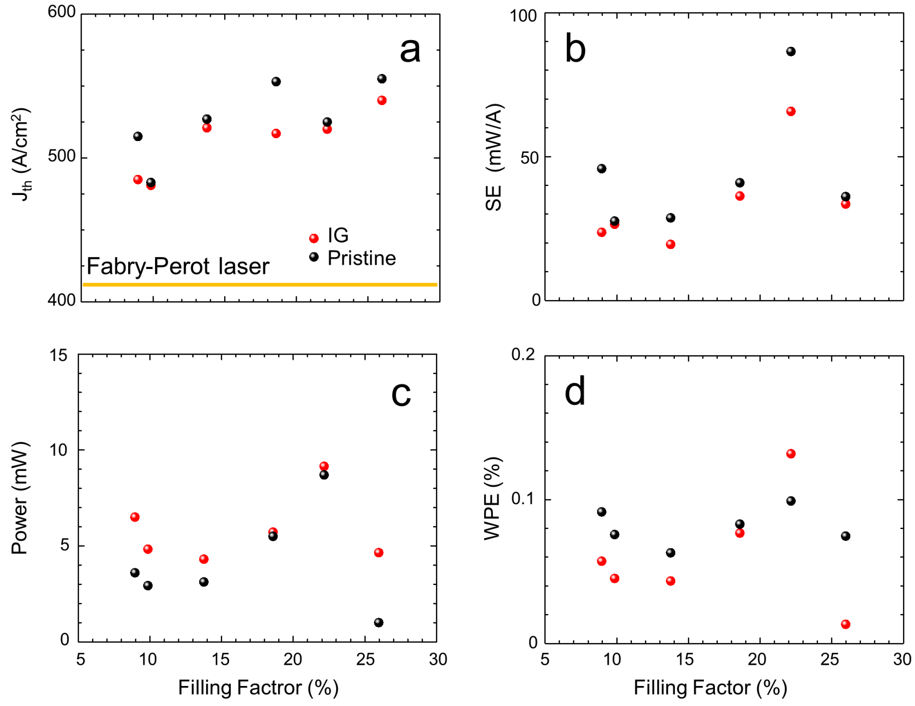

Figure S17. Optical Performance. Figures of merit extracted from several samples comprising one pristine laser (black) and its IG counterpart (red), having filling factor r/a ranging from 8% to 26%. **(a)** Experimental J_{th} extracted from the L-J-V curves. **(b)** Slope efficiency. **(c)** Optical power measured at a heat-sink temperature of 18 K while driving the lasers in quasi-CW mode with a pulse width of 100 μs and a repetition rate of 10 kHz (50%-duty cycle) in a vacuum environment. **(d)** Maximum wall-plug efficiency, calculated as $Pout/Imax * Vmax$, where $Pout$ is the peak optical Power, $Imax$ and $Vmax$ are, respectively, the bias current and voltage corresponding to the maximum $Pout$.

VI. Near-field nanoscopy experiment

The performances of the RL QCL for mapping the THz response of nanosize samples with sub-diffraction spatial resolution are tested by coupling the RL to a commercial near-field scattering-type scanning near-field microscope (s-SNOM) (Neaspec/Attocube). In the experimental setup, sketched in Fig. S18a, the emission of the random QCL is collimated with a 90° off-axis parabolic (OAP) mirror with *f*=50mm focal length, and focused on the AFM tip of the s-SNOM with a 60° OAP with *f*=50mm. The light backscattered by the AFM tip, while approached to the sample, is fed back to the QCL along the same optical path, and detected by monitoring the laser contact voltage exploiting self-mixing interferometry.[9-10] The relative phase between emitted field and optical feedback, which contains the near-field information, is varied with an optical delay line consisting of two 45° plane mirrors mounted on a motorized linear stage (Physik Instrumente) with resolution 0.1μm, that modify the length of the external cavity formed by the QCL surface and by the AFM tip. This latter (RMN 25PtIr200B-H) is operating in tapping mode at frequency Ω_t= 79 kHz such that the near-field component of the backscattered field is isolated from the far-field background by lock-in detection at the harmonics Ω_n = n Ω_t of the tapping frequency, where n=1,2,…5 is the

harmonics order. The near-field self-mixing signal at the n-th harmonics $S_n = s_n \times \exp(i\phi_n)$ is determined from the measured amplitude s_n and phase ϕ_n. In order to exploit the coherence properties of the random QCL, we simultaneously monitor the BN with a wire-based optical antenna, placed in proximity to the cryostat, and measure the intermode BN signal radiated in the free space by the random QCL.

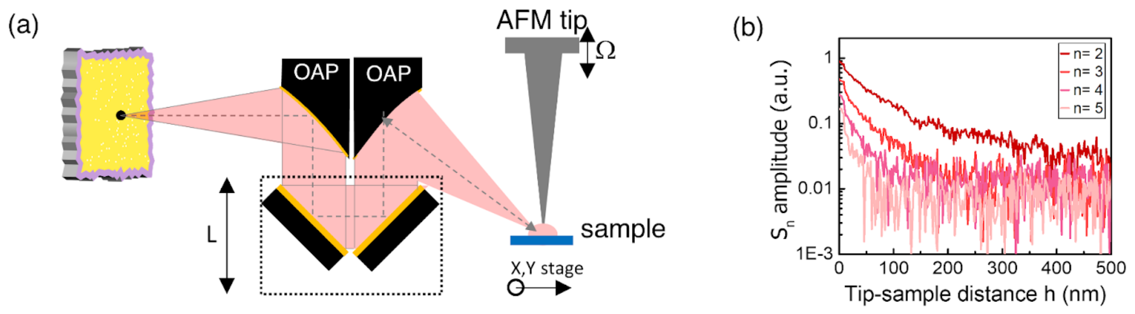

Figure S18. Near-field THz nanoscopy application. (a) Sketch of the near-field scattering nanoscope employing the random QCL as source and detector simultaneously. **(b)** Approach curves measured on a 40nm gold marker evaporated on a 300micron undoped Si substrate, at different demodulation orders n, while operating the random QCL at I=620 mA and T=22K.

The near-field nature of the detected signal is proven by the exponential decay of the harmonics signal observed while we increase the tip-sample distance, see the approach curves in Fig.17b. The lowest order harmonics, reaching the noise level in Fig. S17b is n=2, such that for n>2 we can consider the maps as dominated by the near-field contribution.

a. Near-field maps at different harmonics

By raster scanning, while keeping fixed external cavity length L, we reconstruct the near-field images of a Si static random access memory (SRAM) test sample from Bruker with pre-characterized sub-micrometer regions of controlled p- (PMOS) and n-doping (NMOS) that are expected to exhibit contrast to radiation in the range 0.5-3.5THz. The same sample was previously used for testing the performances of THz broadband near-field nanoscope.[11]

In Fig. S19, we report the near-field images of the test samples, achieved while operating the laser at I=620 mA. We observe a signal to noise ratio (SNR) equal to 30, at the third harmonic with 9ms integration time. This allows detection with high contrast up to the fifth harmonic, exceeding the state-of-the-art performances of broadband THz nanoscopes, reporting only the second harmonic.[9] This is a relevant performance improvement, since the higher the harmonics order, the cleaner the signal from the far-field background.

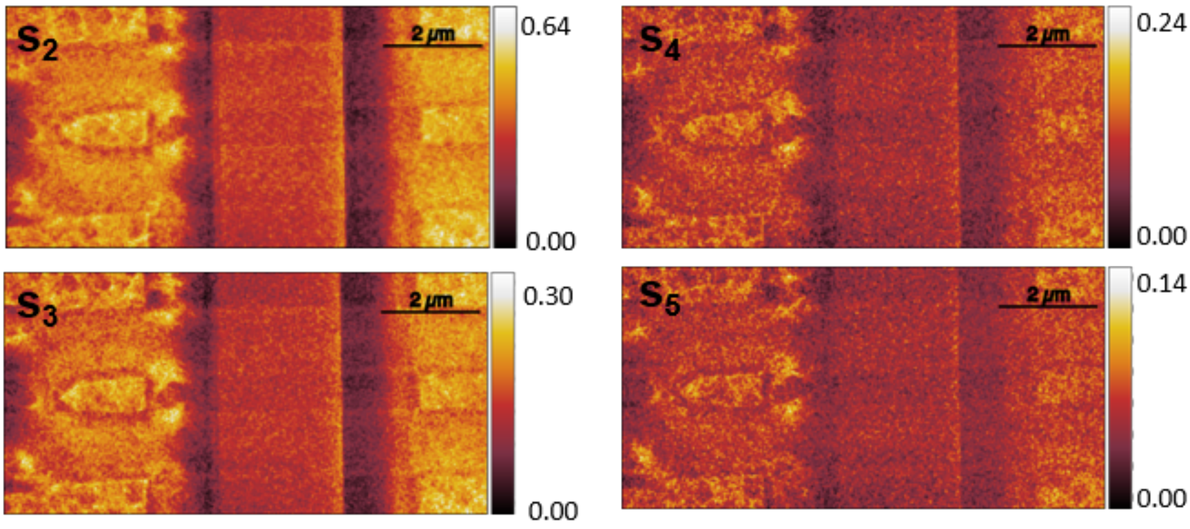

Figure S19. THz near-field maps of a SRAM. (a) Near-field self-mixing maps of the SRAM test sample at different harmonics S_n with n=2,3...5 obtained operating the laser at 620 mA and using 9 ms pixel integration time.

Section VII. Linewidth Modulation in the SMIB experiment

By comparing BN traces acquired at delay line different positions, we observe a ~2 MHz shift in the BN frequency, a ~ 5 time narrowing of the BN linewidth and 10 dB increase of the linewidth power (see Fig.S20). The narrower and more powerful BN, detected by introducing an active modulation of the external optical feedback, corresponds to SNR increase for the self-mixing signal. Both these results rely on the close correlation between phase-locking states of the involved mode and laser feedback, thus confirming mode-locking. The accuracy in the extraction of the BN LWs is limited by the radio frequency (RF) acquisition parameters, chosen to guarantee a stable BN acquisition at each position of the delay line, for the entire duration of the experiment, i.e. ~6h.

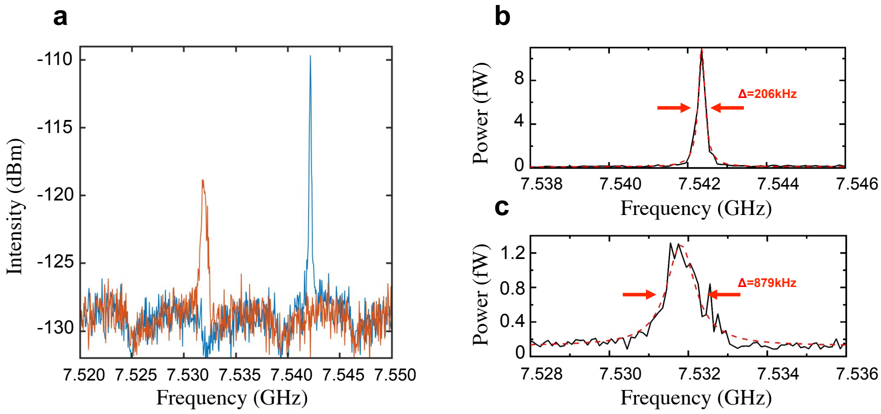

Fig. S20 a. BN spectra acquired at a delay line position of 840 µm (blue curve) and 864 µm (red curve). A 10dB, 400 % changes in power and LW occurs for different feedback delays. The BN signal is extracted from the bias line with a bias-tee and recorded with an RF spectrum analyzer (RBW: 100 kHz, video bandwidth (VBW): 100 kHz, sweep time (SWT): 50 ms, RMS acquisition mode). **b**. Lorentzian fit of the BN acquired at 840 µm, in linear scale, yielding a FWHM of 206kHz. c. Lorentzian fit of the BN acquired at 864 µm, in linear scale, yielding a FWHM of 879kHz, i.e. a~4.27times increase compared to the previous position. The accuracy in the extraction of the BN LWs is limited by the RF acquisition parameters, chosen to guarantee a stable BN acquisition at each position of the delay line, for the entire duration of the experiment~6h.

We then perform SMIB on the same IG laser of Fig.3 of the main text, while driving the laser at a current (720 mA), corresponding to a broad (~14 MHz) BN and no mode locking. In this case, it is not possible to retrieve a noiseless interferometric trace (Fig.S21), comprising overlapping of sinusoidal curves. The linewidth modulation induced by the EOF does not occur only for some specific frequency variations of the BN. The BN central frequency, linewidth and power do not show an ordered behavior as a function of EOF modulation by means of optical path variation.

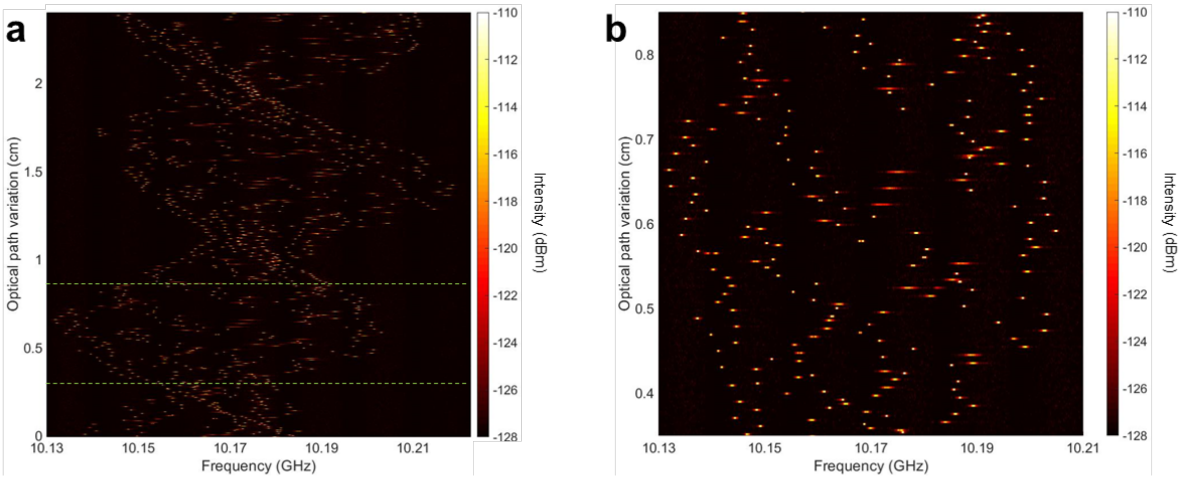

Figure S21: **a**. 2.4 cm long SMIB trace of the BN centered ~10.18 GHz, showing a disordered variation of both frequency shift and linewidth. **b**. Zoom over the section of (a) delimited by the dashed green lines.

Section VIII. Injection Locking in the IG random QCL

To further prove mode locking, we investigate the injection locking dynamics on the IG device, presented in Fig.3 of the main text. We retrieve the beat note spectra as the injected RF power is increased, while varying the RF around the ~12GHz frequency of the single BN. The single BN is pulled towards the frequency of the injected signal (11.95 GHz, Fig.S22a; 11.97 GHz, Fig.S22(b) and finally locked. The simultaneous appearance of a single sideband, ~10 dBm weaker than the initial BN, is also typical of injection locking. [12,13] It persists for RF injected powers from 10-15 dBm up to 35-40 dBm, then the microwave spectrum of the intermode beating is fully controlled by the injected signal. The noise floor around the locked narrow BN is ~-50 dB, i.e., weaker than the peak power of the original BN. This proves that the intermode BN power is almost completely locked. The corresponding locking range (Fig. S22c), as a function of the injected RF follows the square root behavior predicted by Adler's equation. [14]

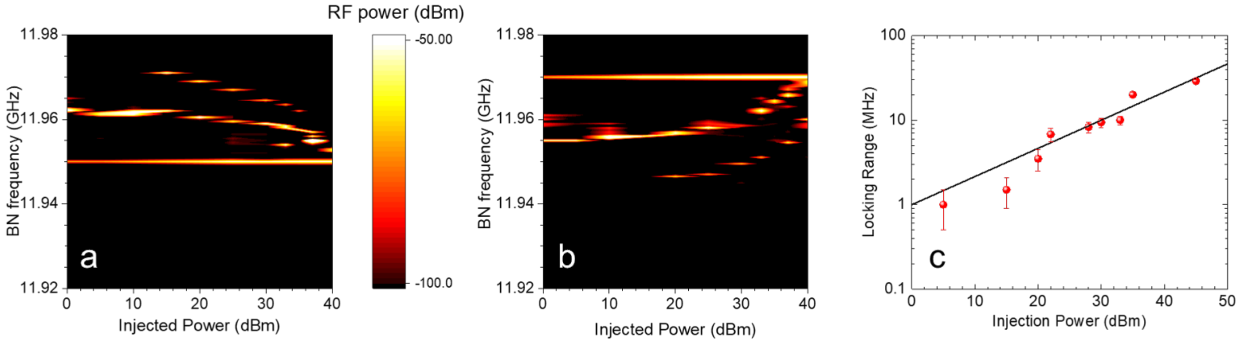

Figure S22. Injection locking experiment on IG Random Laser. (a,b) Intermode BN spectra for different RF injected powers, on the IG random laser of Fig.3 of the manuscript at a bias current of 680 mA. The RF frequency is held constant at (a) +10 MHz and (b) -10 MHz apart from the single BN frequency~11.96 GHz. (c) Locking range as a function of RF-power injected inside the random laser. By directly measuring the RF power transmitted to the laser chip, we estimate a total RF attenuation of ~65 dB.

References

1. Biasco, S. et al. Highly efficient surface-emitting semiconductor lasers exploiting quasi-crystalline distributed feedback photonic patterns. *Light Sci. Appl*. **9**, 1–11 (2020).
2. R., N. R. et al. Fine Structure Constant Defines Visual Transparency of Graphene. *Science* (80). **320**, 1308 (2008).
3. Bonaccorso, F., Sun, Z., Hasan, T. & Ferrari, A. C. Graphene photonics and optoelectronics. *Nat. Photonics* **4**, 611–622 (2010).
4. Lidorikis, E. & Ferrari, A. C. Photonics with Multiwall Carbon Nanotube Arrays. *ACS Nano* **3**, 1238–1248 (2009).
5. Mak, K. F., Ju, L., Wang, F. & Heinz, T. F. Optical spectroscopy of graphene: From the far infrared to the ultraviolet. *Solid State Commun*. **152**, 1341–1349 (2012).
6. Dawlaty, J. M. et al. Measurement of the optical absorption spectra of epitaxial graphene from terahertz to visible. *Appl. Phys. Lett.* **93**, 131905 (2008).
7. Horng, J. et al. Drude conductivity of Dirac fermions in graphene. *Phys. Rev. B* **83**, 165113 (2011).
8. Bianchi, V. et al. Terahertz saturable absorbers from liquid phase exfoliation of graphite. *Nat. Commun*. **8**, 1–9 (2017).
9. Giordano M. C. et al Phase-resolved terahertz self-detection near-field microscopy *Opt. Exp*. **14**, 18423 (2018).
10. Reichel K. C. et al. Self-mixing interferometry and near-field nanoscopy in quantum cascade random lasers at terahertz frequencies. *Nanophoton.* **10**, 1495 (2021).
11. Agharmiri N. A. et al. Hyperspectral time-domain terahertz nano-imaging. *Opt. Exp*. **27**, 24231 (2019).
12. Di Gaspare, A., et al., "Homogeneous quantum cascade lasers operating as terahertz frequency combs over their entire operational regime" *Nanophotonics*, vol. **10**, no. 1, pp. 181-186 (2021).
13. Hillbrand, J., Andrews, A.M., Detz, H. et al. Coherent injection locking of quantum cascade laser frequency combs. *Nature Photon* **13**, 101–104 (2019).
14. R. Adler, "A study of locking phenomena in oscillators," *Proc. IEEE*, vol. **61**, pp. 1380–1385, (1973).
